# Predicting heterogeneity in clone-specific therapeutic vulnerabilities using single-cell transcriptomic signatures

**DOI:** 10.1186/s13073-021-01000-y

**Published:** 2021-12-16

**Authors:** Chayaporn Suphavilai, Shumei Chia, Ankur Sharma, Lorna Tu, Rafael Peres Da Silva, Aanchal Mongia, Ramanuj DasGupta, Niranjan Nagarajan

**Affiliations:** 1 Genome Institute of Singapore, A*STAR, Singapore, Singapore; 2 Department of Physics and Astronomy, University of British Columbia, Vancouver, British Columbia Canada; 3 School of Computing, National University of Singapore, Singapore, Singapore; 4 Department of Computer Science and Engineering, Indraprastha Institute of Information Technology, Delhi, India; 5 Yong Loo Lin School of Medicine, National University of Singapore, Singapore, Singapore

**Keywords:** Drug response prediction, Single-cell RNA-seq, Tumor heterogeneity, Recommender system, Combinatorial therapy

## Abstract

While understanding molecular heterogeneity across patients underpins precision oncology, there is increasing appreciation for taking intra-tumor heterogeneity into account. Based on large-scale analysis of cancer omics datasets, we highlight the importance of intra-tumor transcriptomic heterogeneity (ITTH) for predicting clinical outcomes. Leveraging single-cell RNA-seq (scRNA-seq) with a recommender system (CaDRReS-Sc), we show that heterogeneous gene-expression signatures can predict drug response with high accuracy (80%). Using patient-proximal cell lines, we established the validity of CaDRReS-Sc’s monotherapy (Pearson *r*>0.6) and combinatorial predictions targeting clone-specific vulnerabilities (>10% improvement). Applying CaDRReS-Sc to rapidly expanding scRNA-seq compendiums can serve as in silico screen to accelerate drug-repurposing studies. Availability: https://github.com/CSB5/CaDRReS-Sc.

**Supplementary Information:**

The online version contains supplementary material available at 10.1186/s13073-021-01000-y.

## Background

Tumors comprise heterogeneous populations of malignant cells that display cellular plasticity and phenotypic heterogeneity, as determined by genetic and environmental cues [1–3]. Phenotypic heterogeneity in cancer cells is defined by transcriptomic signatures that govern cell biological behaviors, such as proliferation, apoptosis, migration, invasion, metabolism, and immune response [4, 5]. Intra-tumor transcriptomic heterogeneity (ITTH) can confer differential selective advantages to influence tumor progression and metastasis in vivo [6, 7], as well drug response in vitro [8, 9].

Advances in high-throughput sequencing have enabled large-scale studies into inter-patient tumor heterogeneity at the molecular level [10–12], serving as the basis to distinguish cancer subtypes, investigate tumor biology, and define treatment regimens [2, 13]. These efforts have been complemented by studies on cancer cell lines [14–16] to understand the relationship between molecular markers and drug response in vitro. Several machine learning models have been proposed to utilize information from multi-omic profiles to predict drug response for cell lines [17–23], although significant challenges remain in terms of robustness, generalizability, and translatability into the clinic. In particular, existing models do not explicitly account for intra-tumor transcriptomic heterogeneity and have primarily been trained and tested on clonal cell lines.

In this work, we begin by highlighting the impact of intra-tumor transcriptomic heterogeneity on clinical outcomes based on large-scale re-analysis of TCGA data [24, 25]. We then develop a machine learning framework (Cancer Drug Response prediction using a Recommender System for single-cell RNA-seq or CaDDReS-Sc) that robustly combines single-cell RNA-sequencing (scRNA-seq) data with matrix factorization techniques for recommender system [18, 26] to predict drug response for a heterogeneous tumor. ScRNA-seq data from 12 patient-derived cell lines (PDCs) and cell viability measurements in response to 8 drugs under 2 doses was used to validate CaDDReS-Sc predictions to intra-tumor transcriptomic heterogeneity. Extending to combinations of drugs, we show that drug pairs identified in silico by CaDDReS-Sc to optimally inhibit transcriptionally distinct cell clusters were more effective than individual drugs in vitro.

## Methods

### Datasets and preprocessing

#### Tumor data

Gene expression (FPKM-UQ normalized RNA-seq) and patient survival data for 10,956 tumors from 33 cancer types were obtained from The Cancer Genome Atlas (TCGA) Research Network (https://www.cancer.gov/tcga) [24]. Clinical drug response information and *Response Evaluation Criteria in Solid Tumors* (RECIST) values were obtained from prior work to curate drug response information [25], and statistical analysis was limited to drugs with a sufficient number of patients with clinical data (*n*=8 with ≥15 patients in “Complete Response” and “Clinical Progressive Disease” classes).

#### Cancer cell line data for model training

Drug response data and RMA-normalized gene expression data for 1074 cancer cell lines and 226 drugs were obtained from the Genomics of Drug Sensitivity in Cancer (GDSC) database [15] to be used for model training. For model training, only drugs tested at 9 different dosages were used for robust dose-response curve fitting and obtaining half-maximal inhibitory concentrations (IC50) values based on Bayesian sigmoid curve fitting estimates [18]. This selection also prevents the situation where the same drug is tested at different dosage ranges. Following the classification strategy used in the GDSC study [15], cell lines were labeled as sensitive if IC50 values were lower than the maximum dosage used in the experiment and otherwise labeled insensitive. For each gene, log_2_ expression fold-change was calculated with respect to its average expression across cell lines, and cell line kernel features were calculated using Pearson correlation based on 1856 essential genes [18, 27].

#### Single-cell RNA-seq data

Single-cell RNA-seq data for 1241 cells from 12 head and neck patient-derived cell lines was obtained based on a previously published study [8]. Read counts per gene were obtained by mapping reads with STAR (v2.5.2a, default parameters) [28], followed by RSEM analysis (v1.3.0, default parameters) [29]. Cells with <10,000 reads and a cell with a large number of expressed genes (*n*=14,558) were removed (the median number of genes per cell is 7379; Additional file 1: Fig. S1). Genes expressed in <5% of cells were then filtered out to obtain expression values for 15,144 genes from 1171 cells that were used for further analysis. The read count data for all cells was normalized (as TPM values; Additional file :2: Table S1) and used for clustering analysis and drug response prediction. An additional scRNA-seq dataset containing 5902 cells (TPM values for 23,686 genes) from 21 head and neck cancer tumors was also obtained [4]. This additional dataset was only used for evaluating ITTH scores as there is no drug response information available for it.

#### Single-cell clustering and cluster-specific transcriptomic profiles

A standard scRNA-seq workflow described in the Scanpy tutorial was used to perform single-cell clustering [30, 31]. Starting from the TPM matrix, cells with a large proportion (25%) of mitochondrial genes were removed as the high proportions are indicative of poor-quality cells [32]. Expression values were log-normalized and adjusted based on the detection of highly variable genes. The neighborhood graph was generated with n_neighbors=10 and n_pcs=40. In the final step, the neighborhood graph was used for cell clustering using the Louvain algorithm [33].

To obtain a higher resolution of clustering, subclusters of large clusters (≥ 50 cells) were identified using the same process as the first round clustering. In total, 23 clusters were identified for the Sharma et al. dataset (1171 cells) and 62 clusters for the Puram et al. dataset (5902 cells) (Additional file 3: Table S2-3) [4, 8]. Transcriptomic profiles for each cell cluster (patient) were obtained by averaging TPM values across cells, to be used later for cluster-level (patient-level) drug response prediction.

#### In silico deconvolution and intra-tumor transcriptomic heterogeneity

Percentages of pre-defined cancer cell types in each tumor were identified by using CIBERSORT [34], a tool for tumor deconvolution based on transcriptomic information. Since CIBERSORT requires a cell signature matrix containing gene expression profiles of specific cell types, we followed CIBERSORT’s manual to construct a new signature matrix using GDSC histological subtypes (*n*=53) to obtain a signature matrix with 1529 marker genes.

To measure the degree of heterogeneity for each sample based on the deconvolution result, we defined an intra-tumor transcriptomic heterogeneity score (ITTH) as information entropy of the corresponding cell type profile, i.e., $ITTH=-\sum \limits_i{P}_i\mathit{\log}{P}_i$, where *P*_*i*_ is the fraction of cells with cell type 푖 identified in the tumor (Additional file 4: Table S4-6). Cell types with <5% frequency were excluded to reduce the impact of classification noise and obtain a robust score based on the dominant cell types. For patients with two tumor samples in the TCGA dataset (3.7% of patients), we observed a high correlation of ITTH scores between both tumors of the same patient (Pearson *r*=0.77, *p* value<1.39×10^-7^) and an average ITTH score was used. Patients were classified at the Pan-cancer level into three categories (low, medium, and high) based on the first and third quartiles of ITTH scores. Gene expression profiles for tumors from TCGA were clustered using nonnegative matrix factorization (NMF; *k* = 3 to mimic the number of ITTH clusters) [35].

For scRNA-seq data, a proxy for bulk gene expression values was obtained by calculating the average gene expression across all cells and used to compute ITTH scores as described above. Gold-standard ITTH scores (single-cell ITTH) were then computed based on single-cell clustering (as described above) and then computing information entropy as before, i.e., $-\sum \limits_j{P}_j\mathit{\log}{P}_j$, where *P*_*j*_ is the fraction of cells that belong to cluster *j*.

### The CaDRReS-Sc framework

#### Learning a pharmacogenomic space

A pharmacogenomic space is a latent space that captures the relationship between drugs and samples (transcriptomic profiles for cells, cell clusters, cell lines, or patients), where a dot product between drug and sample vectors captures drug sensitivity. The pharmacogenomic space is learned in CaDRReS-Sc (https://github.com/CSB5/CaDRReS-Sc) [36] based on both transcriptomic and drug response profiles across multiple samples and drugs. The original objective function proposed in [18] was defined as follows:

$$ \operatorname{Minimize}\ \frac{1}{2}\frac{\sum_i{\sum}_u{\left({s}_{iu}-{\hat{s}}_{iu}\right)}^2+\mathrm{regularization}}{\mathrm{K}} $$

$$ {\displaystyle \begin{array}{l}{\hat{\mathrm{s}}}_{\mathrm{u}\mathrm{i}}=\upmu +{b}_i^{\mathrm{Q}}+{b}_{\mathrm{u}}^{\mathrm{P}}+{\boldsymbol{q}}_{\boldsymbol{i}}\cdot {\boldsymbol{p}}_{\boldsymbol{u}}\\ {}=\upmu +{\mathrm{b}}_i^Q+{b}_u^P+{q}_i{\left({\boldsymbol{x}}_{\mathrm{u}}{\boldsymbol{W}}_{\mathrm{p}}\right)}^{\mathrm{T}}\end{array}} $$

where *s*_*iu*_, the observed sensitivity score of sample *u* to drug *i*, is defined by *s*_*iu*_ =  − log_2_(*IC*_50_), ${\hat{s}}_{ui}$is the predicted sensitivity score, *Κ* is the total number of drug-sample pairs, *μ* is the overall mean drug response, ${b}_i^{\mathrm{Q}}$ and ${b}_u^{\mathrm{P}}$ are the bias terms for drug *i* and sample *u*, vectors ***q***_*i*_***,p***_*u*_ ∈ *ℝ*^*f*^ represent drug *i* and sample *u* in the *f*-dimensional latent space, and ***W***_*P*_ ∈ *ℝ*^*d* × *f*^is a transformation matrix that projects transcriptomic kernel features ***x***_*u*_ ∈ *ℝ*^*d*^ for each sample onto the pharmacogenomic space. Based on this objective function, the cell line *u* is sensitive to drug *i* when ***q***_*i*_ and ***p***_*u*_ are near each other in the pharmacogenomic space.

As estimates of ${b}_u^{\mathrm{P}}$ do not accurately capture the true bias of an unseen sample, the bias terms μ and ${b}_u^{\mathrm{P}}$were removed, allowing sample bias to be implicitly captured in ***p***_u_. Furthermore, to reduce noise from extrapolation errors for *IC*_50_ values (Additional file 1: Fig. S2), a logistic weight function was introduced to assign a weight for each sample-drug pair as follows:

$$ {c}_{iu}=\mathit{\min}\left(f\left({s}_{iu},{o}_i,l\right),f\left({\hat{s}}_{iu},{o}_i,l\right)\right) $$

where *f* is a logistic function with slope *l* centered at *o*_*i*_, which is the maximum testing dosage for drug *i*. In insensitive cases, the dose-response curve is extrapolated and *IC*_50_ estimates are higher than the maximum tested dosage. Consequently, if both predicted ${\hat{s}}_{iu}$ and observed *s*_*iu*_ dosages are greater than the maximum dosage, then *c*_*iu*_ is close to 0 and the error relative to the extrapolated *IC*_50_ value is down-weighted. Finally, to obtain a cancer type-specific model, *d*_*u*_ > 1 was defined as a weight of training sample *u* from a given cancer type, enabling the model to focus on accuracy for a subset of training samples. As a result, we obtain the final objective function for learning the pharmacogenomic space, which is calibrated for higher accuracy of drug response prediction based on single-cell transcriptomic profiles.

$$ \operatorname{Minimize}\frac{1}{2}\frac{\sum_i{\sum}_u\left({d}_u{c}_{iu}{\left({s}_{iu}-{\hat{s}}_{iu}\right)}^2\right)+\mathrm{regularization}}{\mathrm{K}} $$

$$ {\hat{\mathrm{s}}}_{\mathrm{u}\mathrm{i}}={b}_i^{\mathrm{Q}}+{\boldsymbol{q}}_{\mathrm{i}}\cdotp {\boldsymbol{p}}_{\mathrm{u}} $$

In this objective function, *c*_*iu*_ allows the model to avoid extrapolation errors in *IC*_50_ values from the dose-response curve fitting step, and *d*_*u*_ guides the model to focus on specific indications. Reducing error in predicted IC50 values allows for the estimation of cell death percentages at specific dosages for both mono- and combinatorial therapy.

#### Model training and evaluation

The CaDRReS-Sc matrix factorization model was trained with a 10-dimensional pharmacogenomic space (*f* = 10), learning rate of 0.01, and maximum number of epochs set to 100,000. All training samples were used for updating the trainable parameters in each epoch (Additional file 1: Fig. S3). Performance on unseen samples was estimated with 5-fold cross-validation within the GDSC dataset, and predictive performance for each drug was measured in terms of prediction accuracy and median absolute error (MAE). Drug-sample pairs were classified into two classes based on their IC50 values, sensitive (IC50 ≤ maximum testing dosage), and insensitive (IC50 > maximum testing dosage) to calculate prediction accuracy. To measure how precisely the model can predict IC50 values, we calculated MAE for each drug-sample pair belonging to the sensitive class.

#### Combining cell-specific drug response values into an overall response value

IC50 values from CaDRReS-Sc’s pharmacogenomic space provide cell-specific information on the dose-response curve that would have to be integrated across cells to get an overall response profile for a patient. In particular, an average of IC50 values (or weighted average for cell clusters) does not take into account the sigmoid shape of the curve, resulting in inaccurate aggregate IC50 values (naïve estimation, Additional file 1: Fig. S4). To improve the accuracy of aggregate IC50 calculations, we employed Newton’s method to iteratively approximate the combined dose-response curve based on cell percentages, individual IC50 values, and estimated slopes (default=1). The naïve estimate was used to start the iterations, which were observed to converge rapidly in practice.

#### Benchmarking drug response predictions for unseen cell types

CaDRReS-Sc was benchmarked against other state-of-the-art machine learning-based approaches for drug response prediction, including ElasticNet [15], cwKBMF [20], SRMF [21], and RWEN [19], based on the GDSC dataset and 5-fold cross-validation. ElasticNet is widely used as a standard model, cwKBMF is a component-wise multiple kernel learning model that outperformed the best performing model from the DREAM challenge [37], SRMF is a collaborative filtering model, and RWEN is a model that aims to reduce the effect of extrapolation errors from the dose-response curve fitting step. For ElasticNet and RWEN, we trained a model for each drug separately based on expression values for all genes. For cwKBMF, we used the same cell line kernel features as CaDRReS-Sc, while excluding drug property information as suggested by the authors. For SRMF, the method does not support prediction for unseen cell lines, as it requires a similarity matrix that consists of both train and test samples. Therefore, we allowed SRMF to use gene expression information for all cell lines but excluded drug response information as appropriate.

#### Predicting drug response for head and neck cancer PDCs

Drugs that elicit a response in at least 30% (13 out of 42) of head and neck cancer cell lines in the GDSC dataset (*n*=81) were used to train a head and neck cancer-specific model (*d*_*u*_ = 10; Additional file 5: Table S7-8). The resulting pharmacogenomic space was used to predict cell, cluster, and patient-specific drug response values (IC50) based on corresponding transcriptomic profiles. IC50 values were used to estimate cell death percentage for a given dosage *o*_*i*_ and aggregated at the patient level for cell (average) and cell cluster (weighted average) predictions.

#### Drug-pathway associations

A pathway activity score was computed as the summation of gene expression log_2_ fold-change values across all genes within each BioCarta pathway [38]. To identify a drug-pathway association, the Pearson correlation was calculated between pathway activity scores and predicted drug response values (cell death percentage) across all training samples. Positive correlation coefficient values indicate that high pathway activity is associated with increased drug sensitivity.

#### Predicting combinatorial therapy response

To predict combinatorial therapy response, predicted cell death percentages *h*_*i*_ and *h*_*j*_ at specific dosages *o*_*i*_ and *o*_*j*_ of drug *i* and *j* for each cell cluster were aggregated for each cluster as *h*_*i*_ + *h*_*j*_ – *h*_*i*_*h*_*j*_, where *h*_*i*_*h*_*j*_ represents the percentage of cells inhibited by both drugs. To estimate response for a patient, the weighted average of cell death percentages was computed across cell clusters.

The potential utility of a drug combination over individual drugs was calculated as the increase in cell death percentage for the combination compared to the best individual drug within the combination. To prioritize drug combinations for the experimental study, we first confirmed that monotherapy predictions showed high cross-validation accuracy, further identified individual drugs that could inhibit different subclones within a patient, and focussed on combinations that were predicted to improve over monotherapy for at least one patient (Additional file 6: Table S9-10).

### Experimental validation

#### Cell line isolation and cell culture

Cell lines were isolated from patients with oral squamous cell carcinoma (OSCC) as mentioned in previous work [9]. Briefly, tumors were minced and enzymatically dissociated using 4 mg/mL-1 Collagenase type IV (Thermo Fisher, cat. no. 17104019) in DMEM/F12, at 37 °C for 1 h. Post digestion, cells were pelleted and resuspended in phosphate-buffered saline (Thermo Fisher, cat. no 14190235) for 3 cycles. Cells were then strained through 70-μm cell strainers (Falcon, cat. no. 352350), prior to pelleting and resuspension in RPMI media (Thermo Fisher, cat. no 61870036), containing 10% fetal bovine serum (Gibco, cat. no 10270-106) and 1% penicillin-streptomycin (Thermo Fisher, cat. no. 15140122). Cells were plated on CellBIND plates (Corning, cat. no 3335) and kept in a humidified atmosphere of 5% CO_2_ at 37 °C. Cells were routinely screened for mycoplasma contamination using MycoAlertTM PLUS Mycoplasma Detection Kit (Lonza, cat. no: LT07-710).

#### Compounds, drug response, and cell viability assays

For each patient, a separate line was isolated from the tumor obtained from the patient’s primary and metastatic lymph node sites. Approximately 5000 cells (2500 primary and 2500 metastatic cells) were seeded per well of a 96-well plate, 24 h prior to drug treatment. Drugs that were used for treatment were obtained from SelleckChem, MedChemExpress, and Cayman Chemical. Docetaxel (cat. no. S1148), Doxorubucin hydrochloride (cat. no. S1208), Epothilone B (cat. no. S1364), Obatoclax Meylate (cat. no. S1057), PHA-793887 (cat. no. S1487), PI-103 (cat. no. S1038), and Vorinostat (cat. no. S1047) were obtained from Selleckchem, while Gefitinib was purchased from Cayman Chemical (cat. no. 13166) and Staurosporin (cat. no. HY-15141) from MedChemExpress. Cells were treated at a drug concentration that corresponds to the median IC50 value for head and neck cancer cell lines seen in the GDSC database [15], as well as at a concentration that is 3-fold lower (Additional file 7: Table S11). All compounds were dissolved in DMSO (Sigma Aldrich, cat. no. D8418) and kept at a constant 1% (v/v) across all drug concentrations and controls. Cells were treated for 72 h prior to the evaluation of drug response. The amount of viable cells post drug treatment was quantitated using CellTiter-Glo luminescent reagent (Promega, cat. no. G7572). An integration time of 250 ms was used when luminescence signals were read using TECAN Infinite M1000 pro-multi-mode plate reader. The relative luminescence of each well was computed using the following formula (Luminescence _Drug_/ Luminescence _DMSO_) and expressed as percentage cell viability (Additional file 7: Table S12-13). The median cell death percentage (100—cell viability) was then calculated across replicates.

## Results

### Intra-tumor transcriptomic heterogeneity is significantly associated with treatment response and patient outcomes

**Fig. 1:**
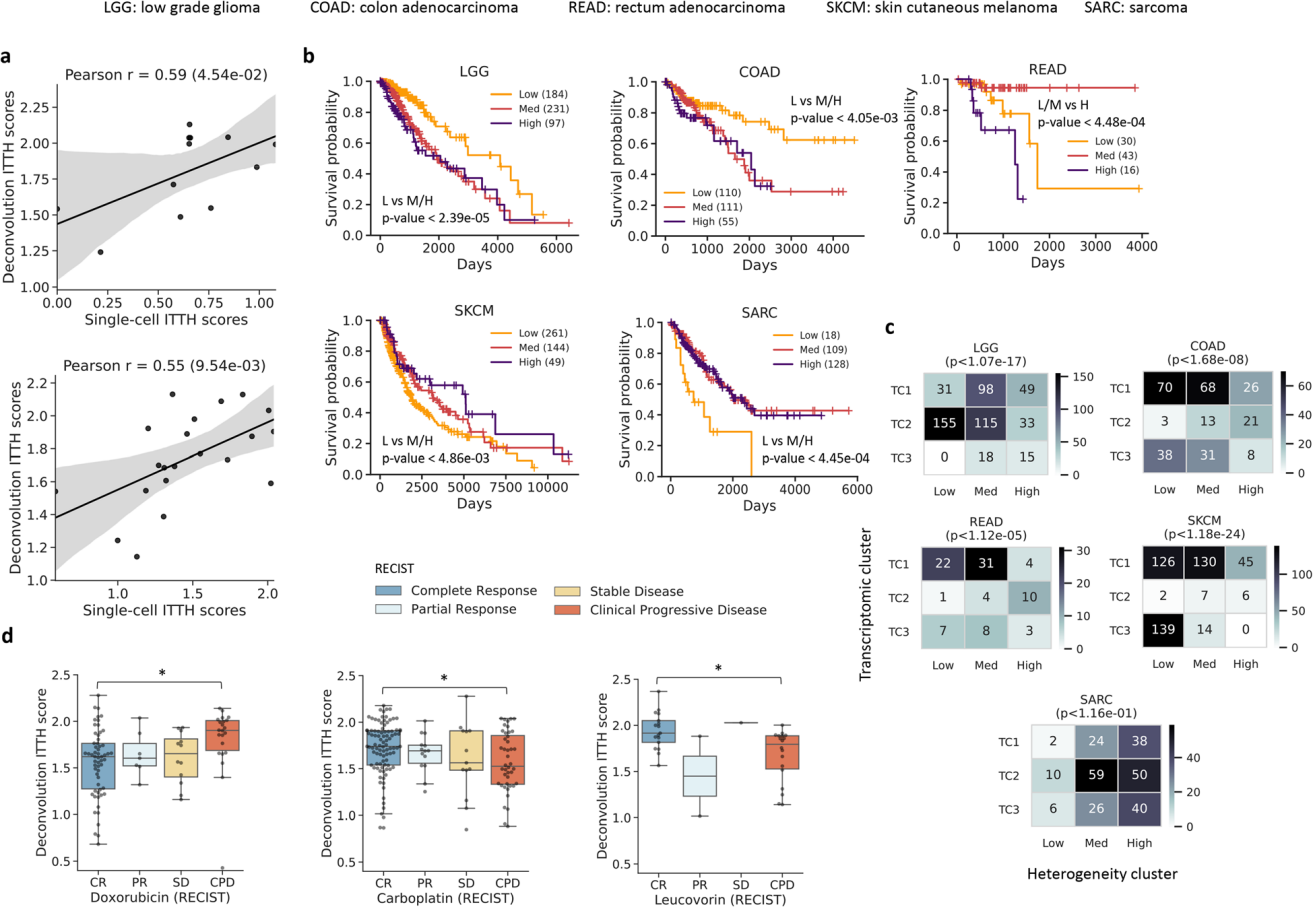
Impact of intra-patient transcriptomic heterogeneity on clinical outcomes. **a** Scatterplots showing the correlation between transcriptomic heterogeneity estimates based on in silico deconvolution (ITTH score) versus single-cell analysis derived values (scITTH score). **b** Survival analysis with ITTH clusters (Low/Medium/High) identified significant differences across various cancer types (FDR-corrected log-rank *p* value<0.05). **c** Plots depicting the overlap between clusters based on transcriptomic profiles (TC) and ITTH scores. *P* values are based on Fisher’s exact test and indicate that the clusters are distinct for most cancer types. **d** Comparison between ITTH scores of patients from different RECIST classes for Doxorubicin, Carboplatin, and Leucovorin highlighting significant differences (^*^FDR-corrected Wilcoxon *p* value<0.05)

To investigate the relationship between intra-tumor transcriptomic heterogeneity (ITTH) and clinical outcomes, we leveraged transcriptomic data from The Cancer Genome Atlas (TCGA) for 10,956 tumors across 33 cancer types, and an in silico deconvolution approach [34], to define a transcriptomic heterogeneity score for each patient (ITTH score, measuring the degree of heterogeneity in gene expression across cells of a tumor inferred based on bulk transcriptomic profiles; Methods). Comparing these in silico heterogeneity scores with single-cell RNA-seq derived gold-standards (single-cell ITTH score—scITTH; Methods) on two different datasets [4, 8] showed that the in silico scores provided a useful proxy to capture transcriptomic heterogeneity (Pearson *r*=0.55 and 0.59; Fig. 1a).

Survival data for various cancer types was then analyzed to detect differences in patients with low, medium, and high transcriptomic heterogeneity (24/33 cancer types with ≥15 samples in each class). Significant associations between ITTH and survival were observed in 5 cancer types, with high heterogeneity associated with poorer outcomes in some cancer types and low heterogeneity in others (Fig. 1b). The most significant associations were observed in low-grade glioma (LGG, Low vs Med/High; FDR-corrected log-rank *p* value<2.39×10^-5^) and sarcoma (SARC, Low vs Med/High, *p* value<4.45×10^-4^), in agreement with prior work on the impact of cell-type diversity in low-grade glioma (LGG) [39] and cellular plasticity in sarcoma (SARC) [40] on treatment outcomes.

To investigate if information in ITTH clusters is captured directly in clustering based on bulk transcriptomic profiles, corresponding clusters were compared for the 5 cancer types (LGG: low-grade glioma, SARC: sarcoma, COAD: colon adenocarcinoma, READ: rectum adenocarcinoma, SKCM: skin cutaneous melanoma; Fig. 1c; Methods). Among these 5 cancer types, associations were observed between transcriptomic clusters and survival in 2 cancer types (LGG, SKCM; FDR-corrected log-rank *p* value<0.05), and these transcriptomic clusters were typically observed to be orthogonal to ITTH clusters (Fig. 1c; 4/5 cancer types; chi-squared test *p* value<0.05). For example, in low-grade glioma, the low ITTH cluster is characterized by a better survival rate compared to transcriptomic clusters 1 and 2 (TC1, TC2), while in rectum adenocarcinoma, the high ITTH cluster is characterized by a lower survival rate compared to all three transcriptomic groups (Additional file 1: Fig. S5), highlighting the additional information captured in ITTH analysis.

Drawing on the availability of patient response data in TCGA for a few drugs (*n*=8) and cancer types (*n*=24), we systematically assessed associations between ITTH scores and clinical drug response (CR: complete response, PR: partial response, SD: stable disease; CPD: clinical progressive disease; Fig. 1d, Additional file 1: Fig. S6). Significant associations were identified in 3/8 drugs (Doxorubicin, Carboplatin, Leucovorin; FDR-corrected Wilcoxon *p* value<0.05; Fig. 1d; Methods), where for example, Doxorubicin-resistant patients exhibited significantly higher transcriptomic heterogeneity (*n*=80; CR vs CPD Wilcoxon *p* value< 9.86×10^-3^). This response pattern for Doxorubicin in patients with high ITTH scores could indicate pre-existing resistant populations [41], tumor evolution [8], or high variability in drug-target engagement [42]. For Carboplatin (Fig. 1d; *n*=138; CR vs CPD Wilcoxon *p* value<2.06×10^-2^), and Leucovorin (*n*=35; CR vs CPD *p* value<2.06×10^-2^), the opposite trend was observed where responders showed significantly higher transcriptomic heterogeneity, consistent with prior work on other platinum compounds such as Cisplatin [43]. Direct measurement and incorporation of transcriptomic heterogeneity could therefore lead to more accurate predictions for drug response, as we explore in the next section.

### Calibrating a recommender system for improved predictive performance on diverse unseen cell types

**Fig. 2:**
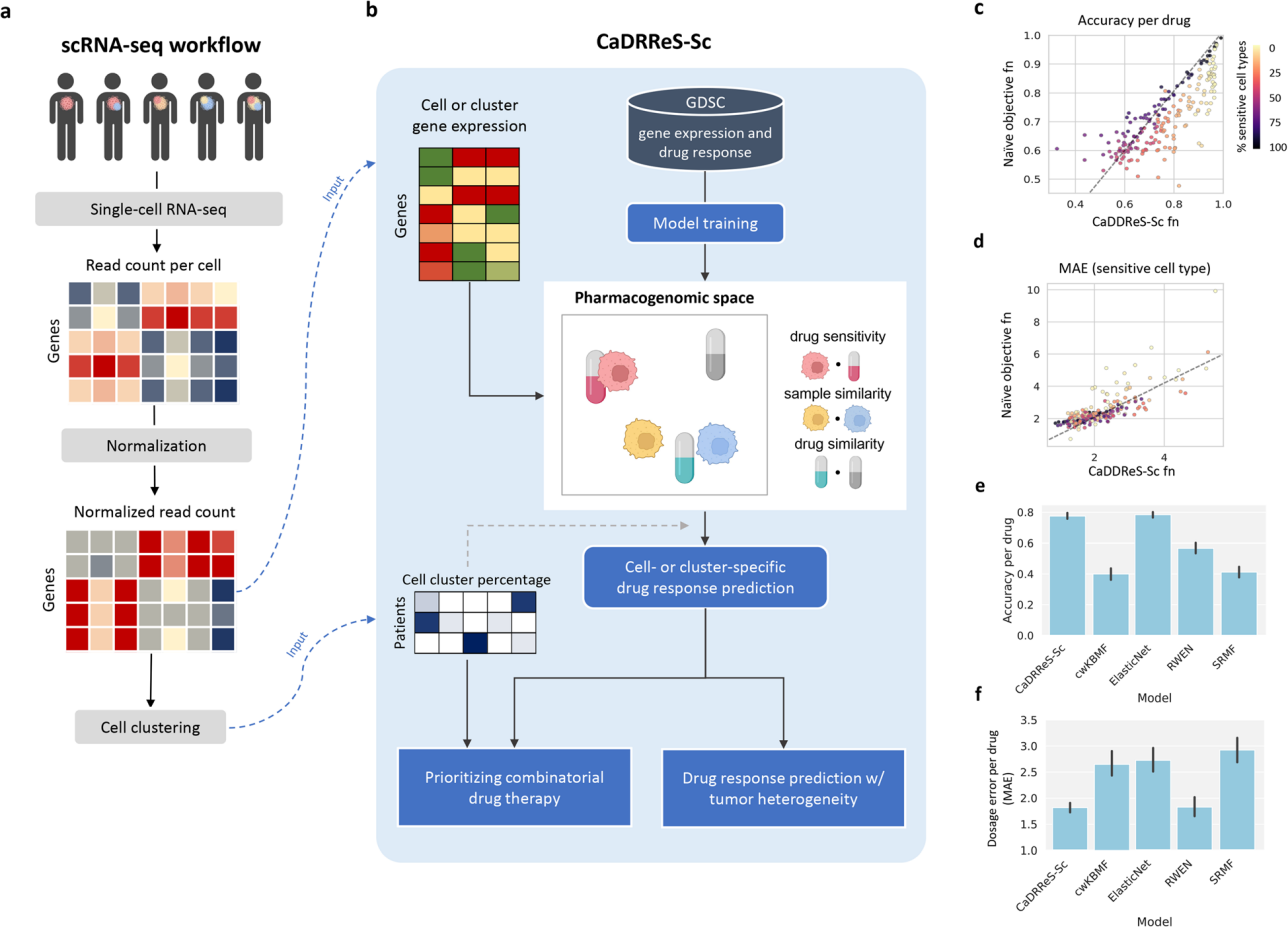
CaDRReS-Sc accurately predicts drug response in unseen cell types. **a** Overview of single-cell RNA-seq workflow to preprocess sequencing data and provide inputs to CaDRReS-Sc (indicated by blue dashed lines). The normalized read count values and cell clustering results are utilized by CaDRReS-Sc for predicting drug response, taking into account transcriptomic heterogeneity within each patient. **b** Overview of CaDRReS-Sc workflow, where a pre-trained *pharmacogenomic space* based on drug response and gene expression profiles from cell-line experiments is used to provide cell- or cluster-specific drug response predictions. These are then combined to estimate overall drug response and prioritize drug combinations for a patient. **c** Comparison of prediction accuracy on unseen cell types between CaDRReS-Sc’s objective function and a naïve function that does not take uncertainty in IC50 values into account. Each dot represents a drug (*n*=226), and dot colors represent the percentage of sensitive cell lines. As can be seen here, CaDRReS-Sc’s objective function is particularly useful when the percentage of sensitive cell lines is low. **d** Comparison of median absolute error (MAE) obtained based on predictions using CaDRReS-Sc as well as a naïve objective function. CaDRReS-Sc’s robust objective function results in lower MAE across a majority of drugs (points above the *y*=*x* line), especially for drugs with a lower percentage of sensitive cell lines (lighter shades). **e** Histograms showing the average prediction accuracy (error bars show 1 standard deviation) using different drug response prediction approaches. **f** Histograms showing MAE (error bars show 1 standard deviation) with different drug response prediction approaches. Overall, CaDRReS-Sc was seen to have high accuracy on the sensitive/non-sensitive classification task while reporting the lowest MAE for the IC50 regression task

The development of single-cell transcriptomics has enabled the direct identification and quantification of cell populations within a tumor [4, 44–46]. Corresponding scRNA-seq workflows with gene expression measurement, normalization, cell clustering, and summarization (Fig. 2a) can be coupled in principle with existing methods that predict drug response from bulk transcriptomic profiles [15, 18–21] to obtain cell-specific response information. However, the utility of such a workflow and potential techniques to obtain a summarized response score for the heterogeneous tumor have not been explored. Besides, a more fundamental challenge is the robustness of such models to diverse, unseen cell types [22].

To address these questions, we develop a machine learning framework trained with cancer cell line data for improved robustness on diverse, unseen cell types, and the ability to combine cell-specific predictions into accurate tumor response values (CaDRReS-Sc; Fig. 2b). Specifically, based on a recommender system technique for cancer drug response prediction [18], we designed a novel objective function that enables the model to simultaneously classify sensitive/insensitive cell types and predict half-maximal inhibitory concentration (IC50) values for sensitive cases (Additional file 1: Fig. S2; Methods). Comparison of predictive accuracy versus a naïve objective function (mean squared error for IC50) on unseen cell lines in the GDSC database showed significant improvements (Fig. 2c; 5-fold cross-validation; Wilcoxon *p* value<2.66×10^-4^), especially for drugs with a smaller proportion of sensitive cell lines. By focusing on predicting response values for sensitive cell lines, we observed that the overall median absolute error (MAE) was significantly reduced in a majority of the drugs (Fig. 2d; 5-fold cross-validation; Wilcoxon *p* value<1.80×10^-5^; Methods), enabling accurate prediction of drug response at specific dosages.

We calculated two different metrics, accuracy to evaluate the ability to differentiate between sensitive and insensitive cell types, and MAE to measure the error of IC50 prediction. The combination of these two metrics allows us to assess overall model performance in providing predictions that can be combined across cell-types for a heterogenous tumor (MAE) and yet provide discriminatory drug response predictions for the drug dosages used in future experiments (accuracy). Benchmarking against other machine learning approaches for drug response prediction trained on the same cancer line dataset, such as ElasticNet [15], cwKBMF [20], SRMF [21], and RWEN [19], we found that average prediction accuracy for CaDRReS-Sc was significantly better than other three methods (cwKBMF, SRMF, RWEN; Wilcoxon *p* value<0.05), with an average prediction accuracy of around 80% compared to <60% for other methods (Fig. 2e, Additional file 1: Fig. S7a). Improvements compared to ElasticNet can be seen in drugs with a smaller fraction of sensitive cell lines, where CaDRReS-Sc’s objective function reduced the adverse effect on the training of limited sensitive cell line data (Additional file 1: Fig. S7b,c). We noted that CaDRReS-Sc, a shared model across multiple drugs (similar to cwKBMF and SRMF), could recapitulate the prediction accuracy of the ElasticNet models that were trained specifically for each drug (Additional file 1: Fig. S8a).

Besides prediction accuracy (i.e., predicting sensitive or insensitive), it is essential to predict precise dosages. Precision in IC50 predictions also allows us to infer responses such as cell death percentage at a given dosage. We observed that by aggregating information across drugs, CaDRReS-Sc showed the lowest prediction error, reducing MAE by >30% compared to cwKBMF, ElasticNet, and SRMF (Fig. 2f). By comparing to the ElasticNet models, we observed that CaDRReS-Sc reduced MAE for most of the drugs and reduced the error by >45% (from median MAE 3.26 to 1.79) for drugs with <50% of sensitive cell types (Additional file 1: Fig. S8b). Finally, we confirmed that a numerical integration-based approach to combine drug response values across cell clusters accurately predicts overall tumor response (Additional file 1: Fig. S4). Together, these capabilities enable CaDRReS-Sc to accurately predict drug response in the presence of transcriptomic heterogeneity as evaluated in the next section based on scRNA-seq data from patient-derived cell lines.

### Accurate drug response prediction in the presence of intra-patient heterogeneity

**Fig. 3:**
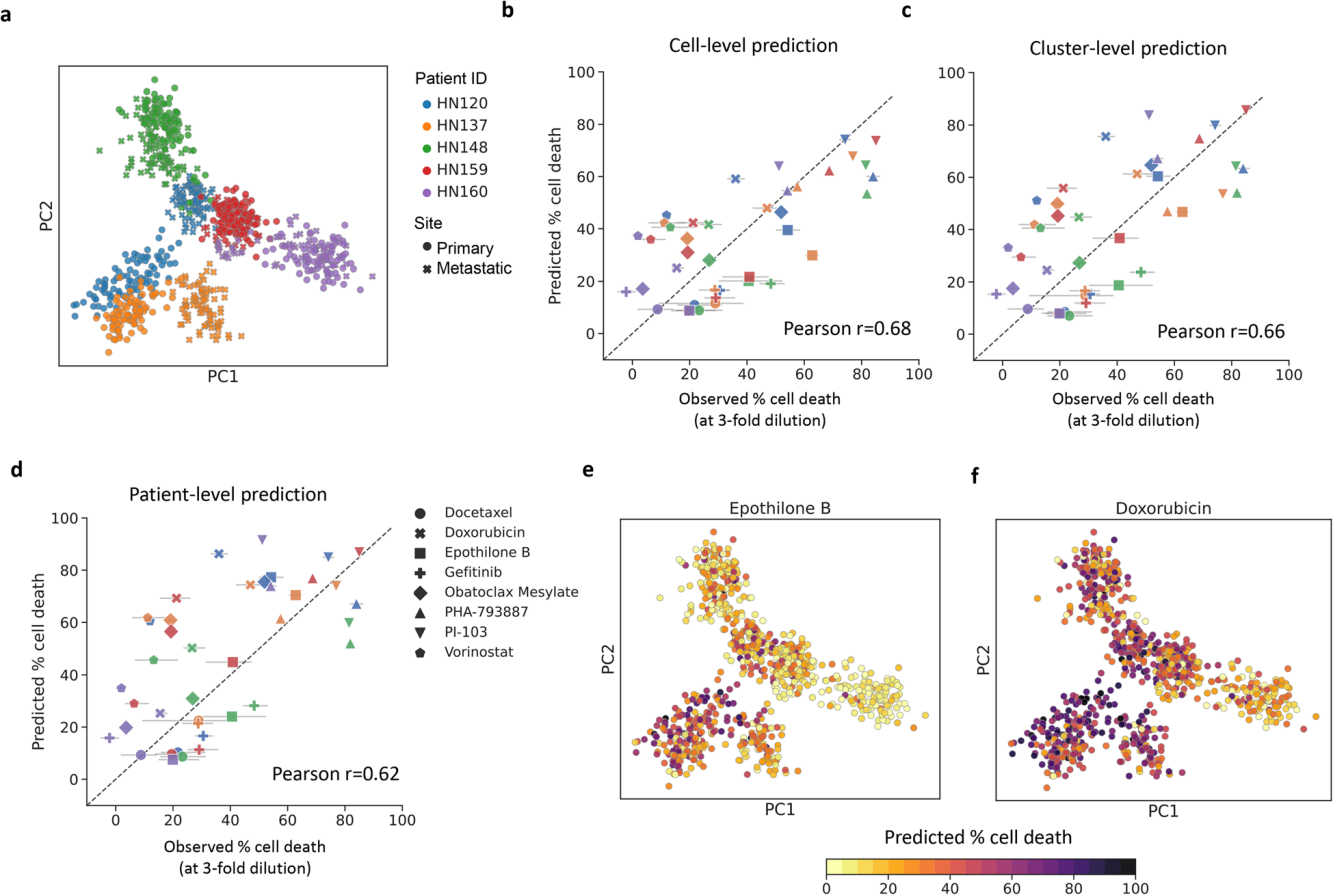
Calibrated drug response prediction in heterogenous patient-derived cell lines using scRNA-seq data. **a** PCA plot showing the diversity of single-cell transcriptomic profiles from different patient-derived cell lines. Comparison of observed and predicted cell death percentages for 5 patient-derived cell lines using 8 different drugs (at lower concentrations), based on CaDRReS-Sc analysis at the **b** cell-level, **c** cluster-level, and **d** patient-level. Error bars show 1 standard deviation based on 3 experimental replicates. Note that that cell and cluster-level predictions show a greater correlation with experimental observations than patient-level predictions, highlighting the utility of scRNA-seq data. **e**–**f** PCA plots showing varied cell-level response predictions to treatment with Epothilone B and Doxorubicin, highlighting substantial inter- and intra-patient drug response heterogeneity

As commonly used cancer cell lines typically lack significant transcriptomic heterogeneity, we leveraged patient-derived cell lines (PDCs) to serve as model systems where sensitivity measurements can be systematically and conveniently made across multiple drugs, while capturing in vivo transcriptomic heterogeneity [9]. In total, 12 PDCs from head and neck cancer patients [8] were used for scRNA-seq analysis (median >10^5^ reads/cell, >5×10^3^ detected genes, >1200 cells in total; Additional file 1: Fig. S1; Methods) and drug response was measured for 8 different drugs at 2 different concentrations (median IC50 of ATCC head and neck cancer cell lines and 3-fold lower; Methods). Visualization of single-cell transcriptomic profiles in 2D space confirmed that significant intra-patient transcriptomic heterogeneity was seen in PDCs (relative to inter-patient heterogeneity; Fig. 3a). Particularly, when primary and lymph node metastatic tumors from HN120 and HN137 are considered, we observed transcriptomically distinct subpopulations that agree with the observations reported in previous studies [8, 9] (Additional file 1: Fig. S9).

We explored several strategies to utilize scRNA-seq data—ranging from using transcriptomic profiles of individual cells, aggregating profiles within a cluster of cells, to combining profiles at the patient-level—for predicting drug response (cell death percentage at a specific drug dosage; Methods). Comparing predictions and drug response observed in our experimental validation (5 pooled PDCs; 8 drugs), we observed significant correlations using CaDRReS-Sc under all three strategies (Fig. 3b, d; Pearson *r*=0.68, 0.66, 0.62, *p* value<1.11×10^-6^, 3.59×10^-6^, and 1.93×10^-5^, respectively; Additional file 1: Fig. S10a-b). We also found that the cell clusters were mapped onto the region near GDSC head and neck cancer cell lines in the pharmacogenomic space (Additional file 1: Fig. S11), suggesting that CaDRReS-Sc could map cell clusters to responses of individual cell lines reported in GDSC. Despite noise and dropout events observed in single-cell data [47], predictions based on cell- and cluster-level transcriptomic profiles consistently showed better agreement with in vitro drug response compared to patient-level prediction (Pearson *r*=0.68/0.66 vs 0.62; consistently across drug dosages; Additional file 1: Fig. S12), highlighting the importance of transcriptomic heterogeneity and the robustness of kernel-based predictions with CaDRReS-Sc (Pearson *r*≤0.59 with ElasticNet and RWEN; Additional file 1: Fig. S13a-b).

As CaDRReS-Sc is based on a *pharmacogenomic space* model that can help interrogate drug-response mechanisms [18], we applied it to our single-cell data to study drug-pathway associations for individual cells (Methods). For example, we found a wide range of responses for Epothilone B (Fig. 3e), especially amongst cells in HN120 and HN137 where primary cells are more sensitive than metastatic cells (Additional file 1: Fig. S14a). Examination of CaDRReS-Sc’s latent pharmacogenomic space identified a significant association between Wnt pathway activation and Epothilone B response (Wilcoxon *p* value<7.24×10^-8^; Additional file 1: Fig. S14c), consistent with prior work on this subject [48]. Similarly, we noted diverse responses across cells for Doxorubicin (Fig. 3f; e.g., primary cells tend to be more sensitive in HN120, Additional file 1: Fig. S14b), and significant association with activation of the Fas pathway (Wilcoxon *p* value<4.69×10^-15^; Additional file 1: Fig. S14d) [49], highlighting the potential to obtain biological insights on therapeutic vulnerabilities based on single-cell information and the interpretability of the CaDRReS-Sc model.

### Drug combinations can be identified in silico by utilizing scRNA-seq data

**Fig. 4:**
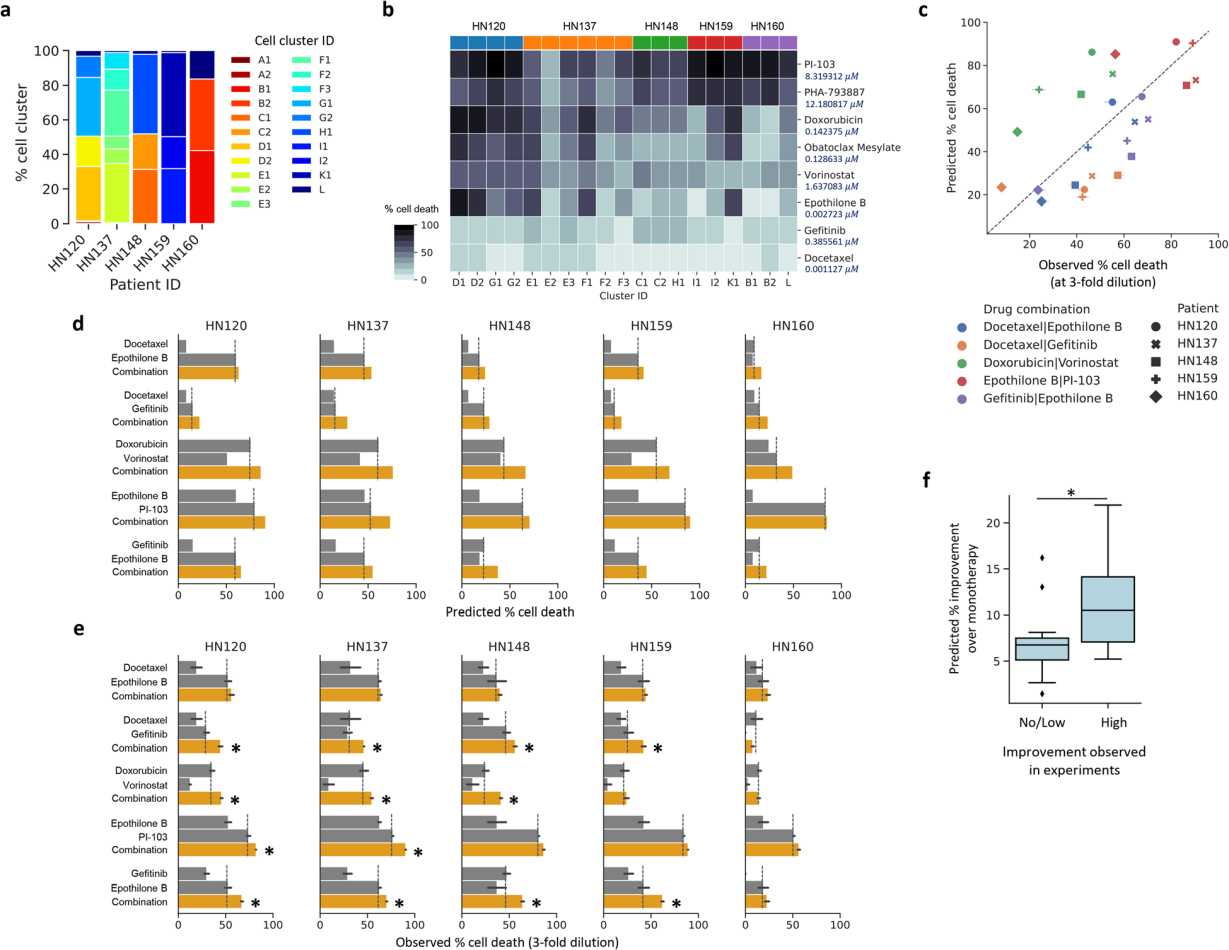
Prioritizing drug combinations targeting transcriptionally-distinct subclones with CaDRReS-Sc. **a** Proportions of various transcriptionally distinct cell clusters (*n*=21) in head and neck cancer patient-derived cell lines. **b** Heatmap of predicted cell death percentages across cell clusters within each patient. **c** Comparison between predicted and observed drug response for five different drug combinations and patient-derived cells. Boxplots contrasting monotherapy (gray) and combinatorial therapy (orange) response based on **d** CaDRReS-Sc predictions and **e** experimental measurements. Error bars show 1 standard deviation (*n*=2–3), dashed lines indicate the best monotherapy, and asterisk symbols indicate drug combinations that show improvement. In general, relative response values for monotherapy and combinatorial therapy, as observed from experimental measurements, were also reflected in CaDRReS-Sc predictions. **f** Boxplots showing that drug combinations that were observed to improve over monotherapy (*x*-axis, no/low vs high determined based on median value in experiment) had significantly higher predicted improvements (combination over monotherapy) using CaDRReS-Sc as well (*y*-axis)

Going beyond monotherapy, the ability to predict drug combinations to target different cancer cell types within a heterogeneous tumor can be essential for improving treatment efficacy in the clinic [50, 51]. The utility of combinations can arise from independent drug action as well as the increased chance of specific clones being sensitive to a drug [52]. To explore this, we evaluated if in silico predictions with scRNA-seq data could reveal drug combinations that provide better response compared to monotherapy, even at lower dosages. Specifically, by inspecting 21 cell clusters across all PDCs identified in our monotherapy analysis, we observed different cluster proportions across patients (Fig. 4a) and a broad range of predicted monotherapy responses across cell clusters (Fig. 4b). These results suggest variability in therapeutic response across different subclones in a given individual, allowing us to identify complementary drug combinations for subclones.

We calculated an expected combination effect (% cell death) for five candidate drug combinations, including Docetaxel:Epothilone B, Docetaxel:Gefitinib, Gefitinib:Epothilone B, Epothilone B:PI-103, and Doxorubicin:Vorinostat, based on predicted cluster-specific drug responses and the distribution of cell clusters. These combinations were then functionally validated on five pooled PDCs via cell-based viability assays using low drug dosages to circumvent off-target effects resulting from extreme inhibition (Methods). Despite the potential for drug interactions [53], CaDRReS-Sc predictions for response to various drug combinations showed a clear correlation to observed responses across the 25 different experimental conditions (Fig. 4c; Pearson *r*=0.58; *p* value<2.30×10^-3^), in comparison to weaker correlations with other methods (Pearson *r*≤0.49 with ElasticNet and RWEN; Additional file 1: Fig. 13c-d).

Testing drugs in each combination at low dosages also helped to mimic what might be needed to support the mitigation of side effects from combinatorial treatment [50]. We then evaluated if this approach can be used to predict pairs of drugs that can elicit greater overall cell death compared to monotherapy. Overall, we observed consistent trends between in silico predictions with CaDRReS-Sc (Fig. 4d) and experimental results (Fig. 4e). For instance, the combination of Doxorubicin and Vorinostat was predicted to provide a notable improvement over monotherapy (+22%) in HN148, which was observed experimentally as well (+11%), consistent with prior work on this combination [54]. By computing the expected improvement of combinatorial therapy over monotherapy, we observed concordance between CaDRReS-Sc’s in silico predictions and in vitro experimental results (Fig. 4f; 25 different experimental conditions; Wilcoxon *p* value<3.39×10^-2^), but no significant associations for other methods (ElasticNet, RWEN). These results indicate that CaDRReS-Sc can sufficiently capture therapeutic response for mono- and combinatorial therapy, enabling prioritization of drugs and combinations for in vitro and in vivo studies.

## Discussion

While the role of intra-patient heterogeneity in genetic mutations has been extensively explored with respect to tumor biology [3, 55], fewer studies have investigated how this combines with epigenetic heterogeneity to influence transcriptomic heterogeneity [39], drug response, and patient outcomes [1, 2]. In this work, we leveraged the availability of large-scale tumor sequencing datasets to highlight the relationship between intra-tumor transcriptomic heterogeneity and patient outcomes, identifying associations in 5 out of 24 cancer types and 3 out of 8 standard-of-care drugs. While this analysis emphasized the general importance of taking ITTH into account for predicting treatment response and outcomes, the power to detect associations might have been limited by the dependence on an in silico deconvolution approach [34, 56]. We also observed that increased ITTH does not always associate with adverse outcomes, as a progressive disease could be associated with convergence to homogeneity (or loss of ITTH) which may influence the response to specific classes of drugs [57, 58]. However, further investigation is needed as the treatment outcome would depend on the MoA of the drug, indication-specific biology, and the distinct vulnerabilities associated with tumor cell populations based on their transcriptome. With the increasing availability of single-cell tumor sequencing datasets, the resolution of such analysis could be greatly improved and help identify shared cell populations that contribute to treatment resistance across patients.

Predicting treatment response in silico in the presence of intra-tumor heterogeneity requires models that provide calibrated values for a single drug across many cell types, while prior work has focused on calibrated predictions for a cell type across many drugs [18]. To address this, CaDRReS-Sc uses a novel objective function that accounts for the uncertainty in drug response values across drugs. This allowed CaDRReS-Sc to train a model that is as accurate as single-drug models (80%), while leveraging information across drugs to provide highly calibrated response values (low MAE) compared to start-of-the-art multi-drug methods. This establishes CaDRReS-Sc as the only method that can differentiate responsive cell types with high accuracy, while minimizing error in computing IC50, for combining into a robust overall prediction for a heterogenous tumor. Furthermore, CaDRReS-Sc’s latent pharmacogenomic model provides ready visualization and interpretation to examine the pathways involved in drug response heterogeneity in a tumor.

Patient-derived cell lines (PDCs) serve as ideal systems for drug sensitivity measurements in vitro while capturing intra-tumor transcriptomic heterogeneity [8, 9], and we leveraged this in a proof-of-concept study, with 12 head and neck cancer PDCs and 8 drugs under 2 dosages, to assess the ability to predict drug response in silico in the presence of transcriptomic heterogeneity. We note that scRNA-seq could have lower coverage compared to bulk RNA-seq and our analysis would be skewed towards highly abundant genes. The Fluidigm C1 chip was used to generate the single-cell data used here, and this yields a relatively higher number of detected genes, allowing drug response prediction to be based on a more representative set of genes.

Several sources of noise in drug response measurement experiments have been reported in previous studies, including lack of experimental replication [14, 15], and discordance in drug response information that was generated based on the same set of cell lines [59]. To mitigate this noise, we trained the model based on one dataset and only utilized drugs that were tested at 9 different dosages to ensure that curve fitting and computed IC50 values would be more reliable. Despite variations in experimental conditions between training data from public cancer cell line datasets [15] and test response data from heterogenous PDCs, in silico predictions from CaDRReS-Sc could recapitulate cell death percentages observed in our in vitro experiments (Pearson *r*=0.68, Fig. 3b), highlighting the robustness of such models. Further availability of drug response data in PDCs and at clinically relevant doses [60] could help advance the predictive performance and clinical utility of such models.

In predicting response to monotherapies, we observed consistently higher correlations with in vitro measurements when using transcriptomic profiles with higher granularity (individual cells or cell clusters versus bulk profiles). This prompted us to consider prioritizing combinatorial therapy options based on CaDRReS-Sc predictions for different subclones, assuming that the combinatorial effect can be approximated in many cases through independent drug action on distinct subclonal cancer cell populations [52]. Independent action of drugs on primary and metastatic tumors with distinct transcriptomic patterns have also been reported [9]. Although this does not directly account for the impact of drug-drug interactions [50, 61, 62], overall, we were able to capture the effect of combinatorial drug therapy (Fig. 4c-e) and its improvement over monotherapy (Fig. 4f) based on distinct drug response profiles across subclones. We envisage, therefore, that the growing corpus of scRNA-seq data can be data-mined using CaDRReS-Sc to identify drug combinations that target clone-specific therapeutic vulnerabilities and lead to better treatment outcomes [8, 41].

## Conclusions

Developing in silico tools for predicting in vivo treatment response remains a challenge as multiple factors (e.g., tumor microenvironment, immune response, overall patient health) can impact patient trajectories. The ability to predict the synergistic effect of multiple drugs on a given sub-population would also be useful information that complements the additive effects across sub-populations that is captured by our framework. In this study, we aimed to bridge the in silico to in vitro gap for predicting response to mono- and combinatorial therapy in the presence of transcriptomic heterogeneity. Together with improved technologies for patient-derived cancer cell models, this combined in silico/in vitro approach could form the basis of a first-cut precision oncology platform that prioritizes mono- and combinatorial therapy options in a clinically relevant timeframe.

## Supplementary Information

**Additional file 1: Fig S1.** Single-cell RNA-seq statistics for 12 patient-derived cell lines. **Fig S2.** Impact of dose-response curves from in vitro cell viability assays on IC50 estimates. **Fig S3.** Training and validation loss. **Fig S4.** CaDRReS-Sc accurately estimates aggregate IC50 values in the presence of transcriptomic heterogeneity. **Fig S5.** Survival analysis for clusters based on bulk transcriptomic profiles. **Fig S6.** Boxplots comparing ITTH scores across clinical response categories for various cancer drugs. **Fig S7.** Additional performance evaluation per drug. **Fig S8.** Pairwise comparison of CaDRReS-SC’s performance on unseen cell types. **Fig S9.** Transcriptomic patterns of cells from HN120 and HN137. **Fig S10.** Detailed comparison between predicted and observed cell death percentages. **Fig S11.** Pharmacogenomic space of GDSC cell lines and HNSC patient-derived cell clusters. **Fig S12.** Comparison of observed and predicted drug response across 5 pooled PDCs and 8 drugs. **Fig S13.** Predictive performance of ElasticNet and RWEN based on cell clusters. **Fig S14.** Comparison of drug response between tumor types and pathway activity groups.

**Additional file 2: Table S1:** TPM values of scRNA-Seq for patient-derived cell lines.

**Additional file 3: Table S2**: Cell clustering results of Sharma et al dataset. **Table S3**: Cell clustering results of Purum et al dataset.

**Additional file 4: Table S4**: ITTH score and drug response. Table S5: CIBERSORT's TCGA deconvolution. **Table S6**: CIBERSORT's signature matrix.

**Additional file 5: Table S7**: GDSC drug statistics. **Table S8**: GDSC cell lines.

**Additional file 6: Table S9**: Drug combinatorial prediction. **Table S10**: Monotherapy prediction.

**Additional file 7: Table S11**: Drug dosages in experimental validation. **Table S12**: Experimental results of monotherapy. **Table S13**: Experimental results of drug combination

## Data Availability

The single-cell RNA-seq data used in this study was previously published in [8], and raw data is available in the Gene Expression Omnibus repository under the series accession GSE117872. All supplementary data, including preprocessed gene expression (scRNA-seq) and experimental drug response of patient-derived cell lines, are available at https://figshare.com/projects/CaDRReS-Sc/75936 [63]. A Python package for CaDRReS-Sc and example scripts for predicting drug response based on scRNA-seq data are available at https://github.com/CSB5/CaDRReS-Sc [36].
