# Supplementary material for: Predicting heterogeneity in clone-specific therapeutic vulnerabilities using single-cell transcriptomic signatures
Source: Genome Med. 2021 Dec 16;13:189. doi: 10.1186/s13073-021-01000-y (PMC8680165; doi:10.1186/s13073-021-01000-y)
Supplement: Supplementary file 1 — Additional file 1: Fig S1. Single-cell RNA-seq statistics for 12 patient-derived cell lines. Fig S2. Impact of dose-response curves from in vitro cell viability assays on IC50 estimates. Fig S3. Training and validation loss. Fig S4. CaDRReS-Sc accurately estimates aggregate IC50 values in the presence of transcriptomic heterogeneity. Fig S5. Survival analysis for clusters based on bulk transcriptomic profiles. Fig S6. Boxplots comparing ITTH scores across clinical response categories for various cancer drugs. Fig S7. Additional performance evaluation per drug. Fig S8. Pairwise comparison of CaDRReS-SC’s performance on unseen cell types. Fig S9. Transcriptomic patterns of cells from HN120 and HN137. Fig S10. Detailed comparison between predicted and observed cell death percentages. Fig S11. Pharmacogenomic space of GDSC cell lines and HNSC patient-derived cell clusters. Fig S12. Comparison of observed and predicted drug response across 5 pooled PDCs and 8 drugs. Fig S13. Predictive performance of ElasticNet and RWEN based on cell clusters. Fig S14. Comparison of drug response between tumor types and pathway activity groups. [file 13073_2021_1000_MOESM1_ESM.pdf]

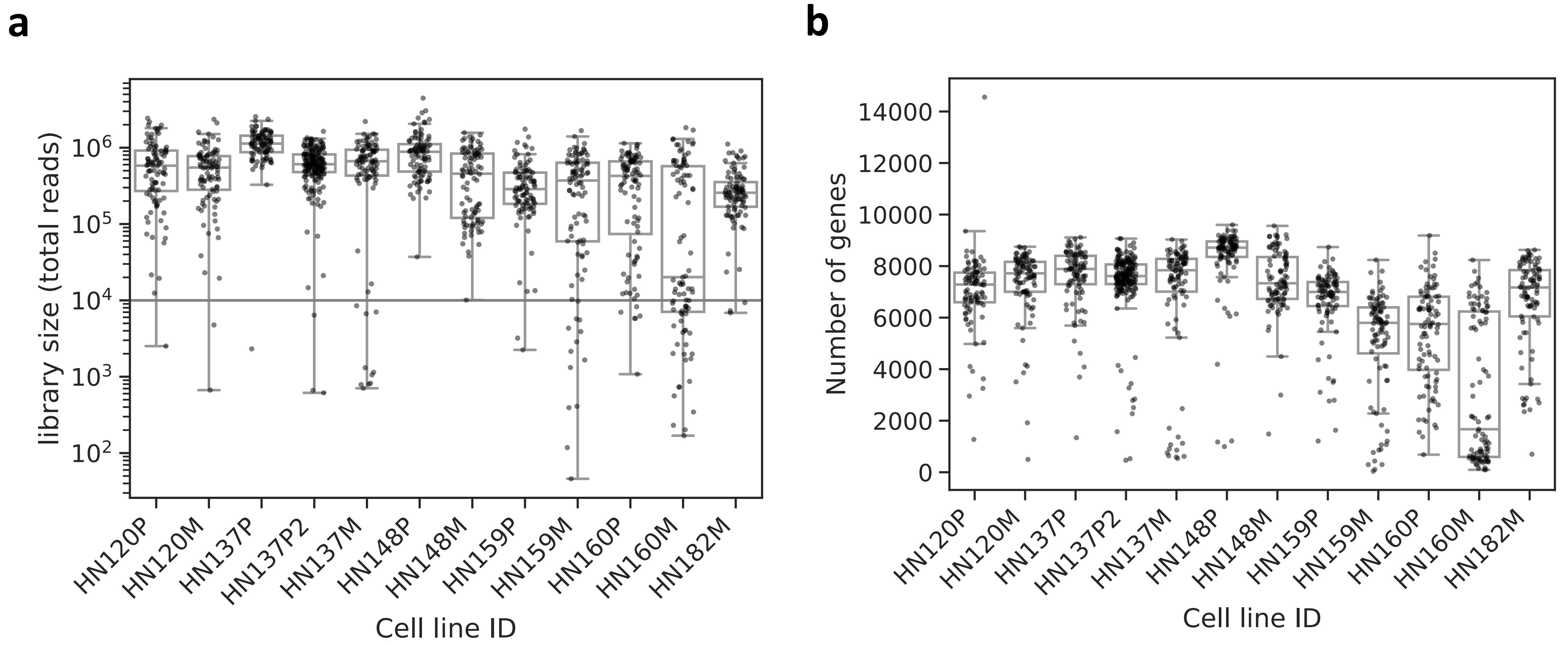

Fig S1: Single-cell RNA-seq statistics for 12 patient-derived cell lines. (**a**) Boxplots depicting total number of reads per cell for the 12 cell lines. (**b**) Boxplots showing total number of expressed genes (read count > 0) per cell for the 12 cell lines.

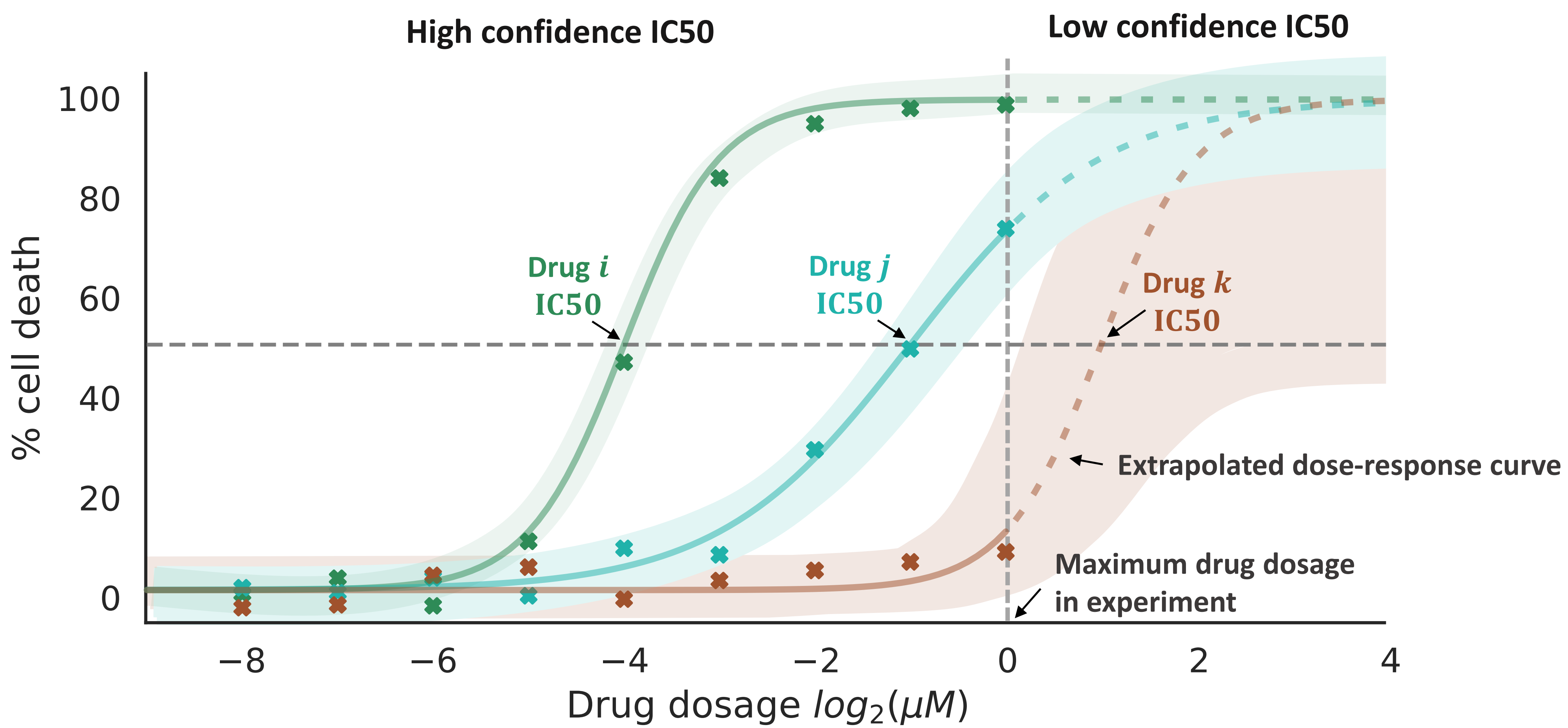

Fig S2: Impact of dose-response curves from *in vitro* cell viability assays on IC50 estimates. Half-maximal inhibitory concentrations or IC50 values are estimated based on fitting a sigmoid function to the dose-response curve. When true IC50 values are beyond the maximum drug dosage tested, this can lead to significant extrapolation noise that impacts model performance. CaDRReS-Sc addresses this issues with a novel objective function that down-weights low confidence IC50 values during model training (see **Methods**).

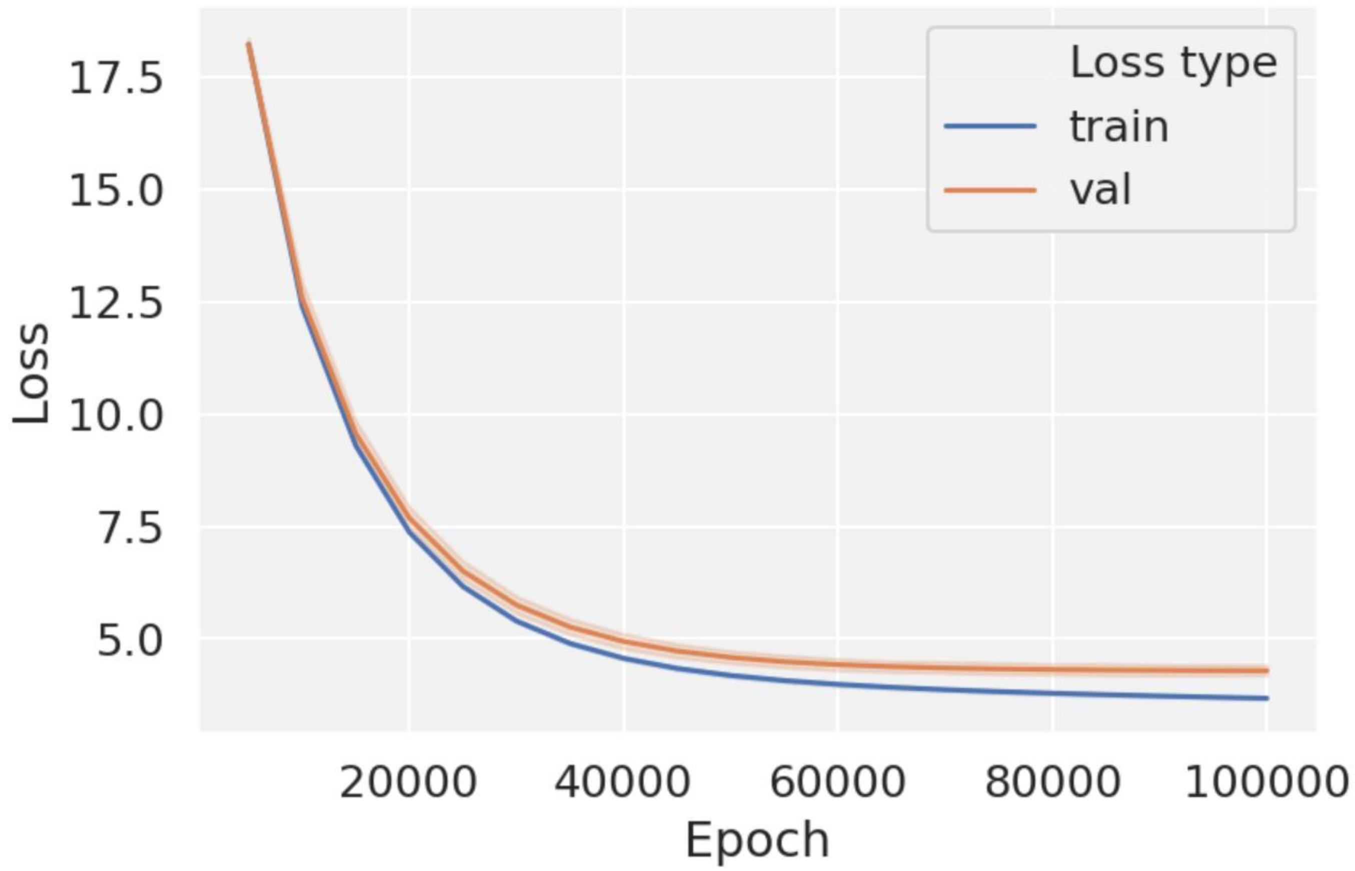

Fig S3: Training and validation loss. In the training step of CaDRReS-Sc (5-fold cross-validation), the model was trained based on all training samples for each epoch, with the maximum number of epochs set to 100,000.

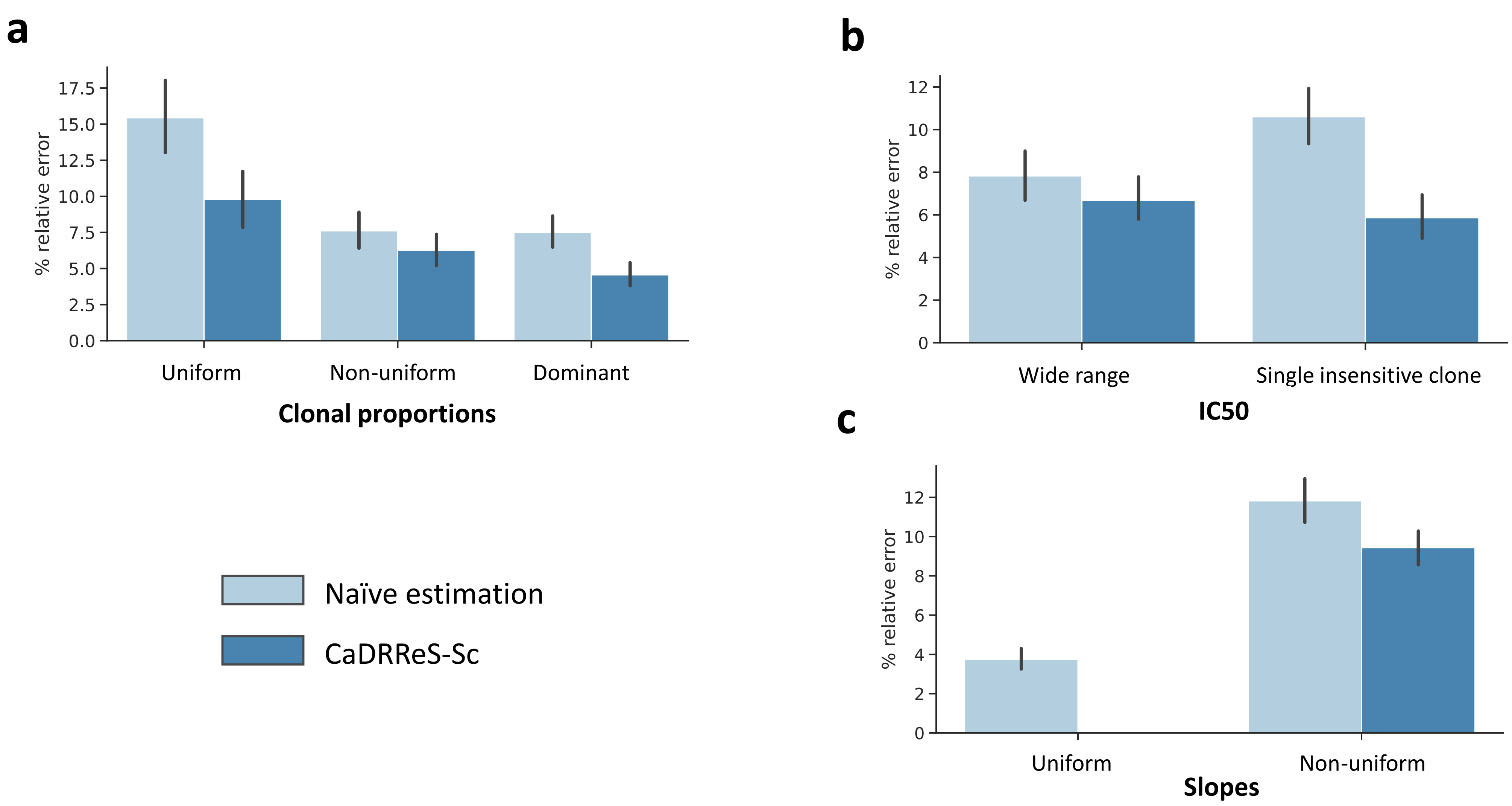

Fig S4: CaDRReS-Sc accurately estimates aggregate IC50 values in the presence of transcriptomic heterogeneity. Simulated samples were constructed with multiple sub-clones (n=[2-10]) with different dose-response curves based on GDSC data (log2 IC50=[1-5] and slope=[1-3]). CaDRReS-Sc estimates (based on numerical integration; see **Methods**) and a naïve estimation (weighted average of IC50 values) were compared to true IC50 values. Based on different conditions for (**a**) clonal proportions (**b**) IC50 values, and (**c**) slopes, CaDRReS-Sc was observed to be more robust compared to the naïve approach. For clonal proportions, *non-uniform* indicates log distribution abundance and the *dominant* case consist of a single dominant clone (80%) and equal proportions of the remaining clones. For IC50, *wide range* (1-5) indicates different IC50s across clones and *single insensitive clone* has a single clone with high IC50 (5) with lower IC50s for the other clones (1). For slopes, *non-uniform* cases refer to the situation with different slopes (1-3) across clones.

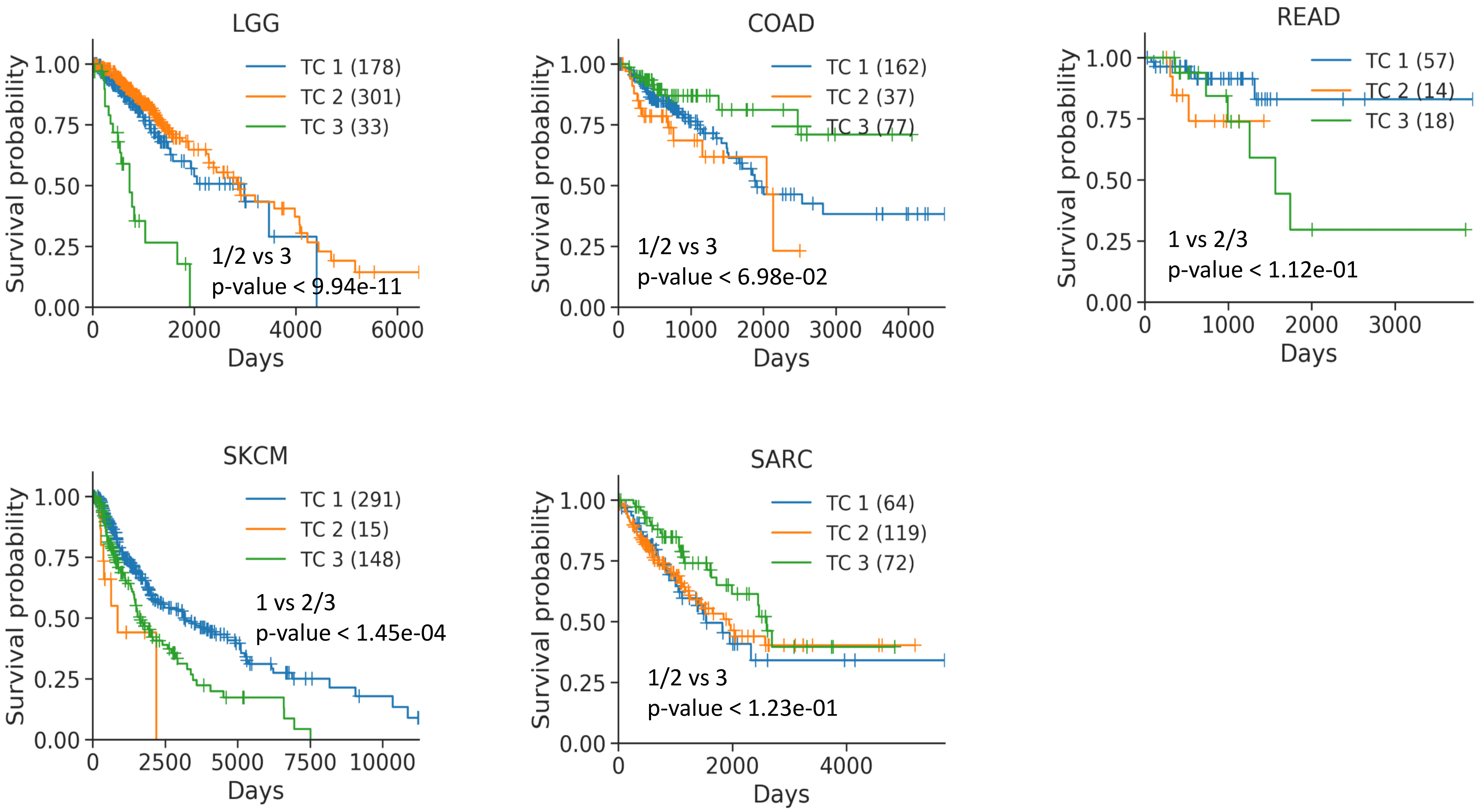

Fig S5: Survival analysis for clusters based on bulk transcriptomic profiles. Note that the figures only show cancer types where significant differences were observed across the transcriptomic clusters (TC). Numbers in parentheses indicate the number of subjects in each cluster. P-values are FDR-corrected and based on the logrank test.

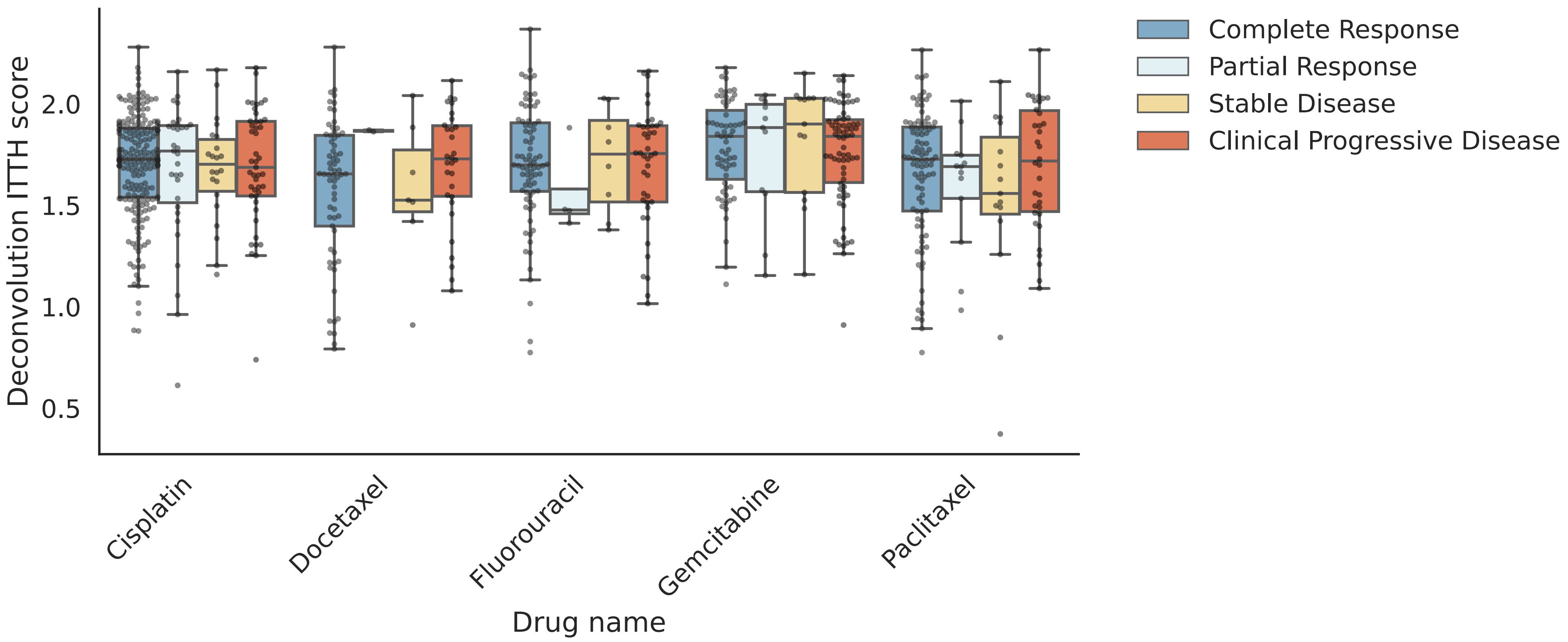

Fig S6: Boxplots comparing ITTH scores across clinical response categories for various cancer drugs. This figure shows drugs that did not exhibit significant differences and the rest are shown in **Figure 1d**.

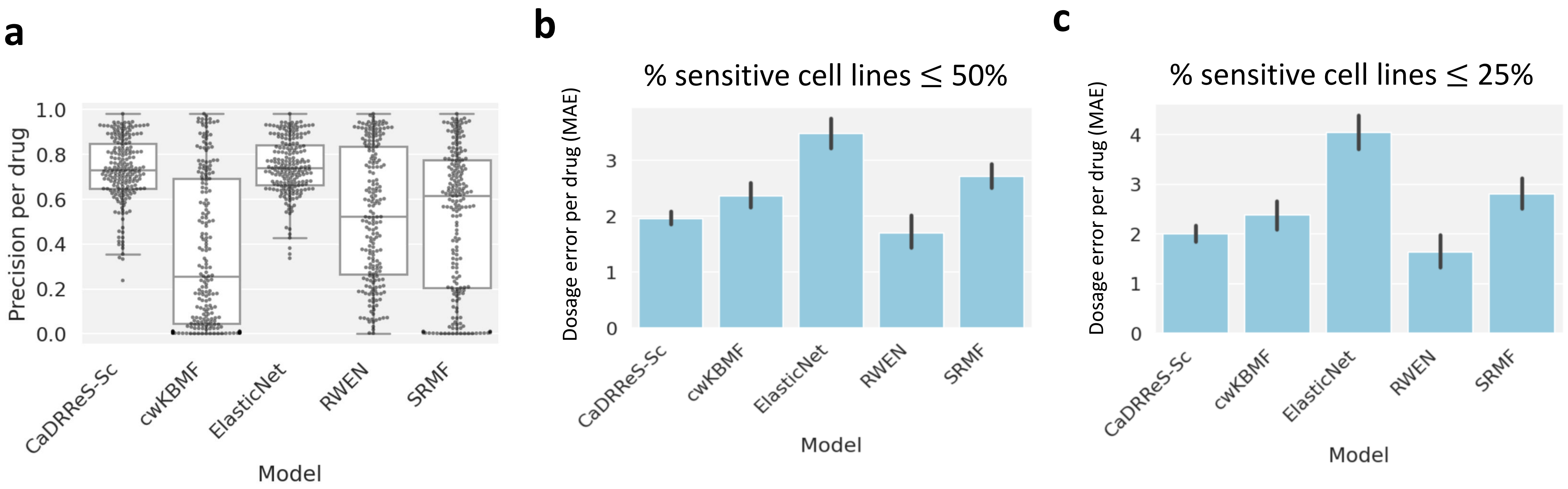

Fig S7: Additional performance evaluation per drug. (**a**) Each dot represents a drug (n=226) with weighted precision (average across all cell lines). For dosage error per drug, Median Absolute Error (MAE) was plotted (**b**) for drugs with ≤50% of sensitive cell lines (n=128) and (**c**) for drugs with ≤25% of sensitive cell lines (n=82).

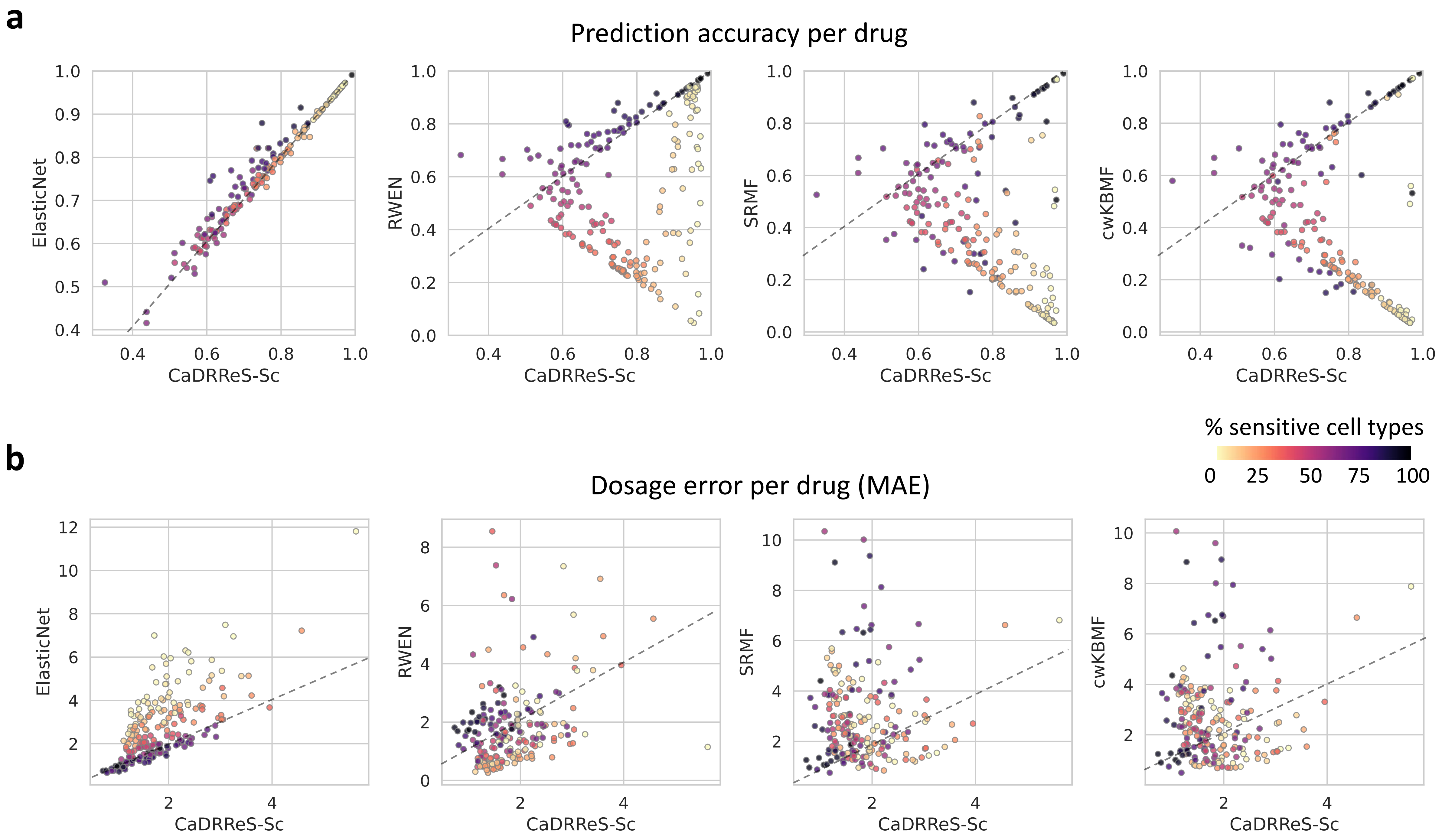

Fig S8: Pairwise comparison of CaDRReS-SC's performance on unseen cell types. Each dot represents a drug (n=226) and dot colors represent the percentage of sensitive cell types. (**a**) Comparison of prediction accuracy, where CaDRReS-Sc typically has higher values. (**b**) Comparison of dosage mean absolute error per drug on a log2 scale, where CaDRReS-Sc typically has lower values compared to other methods.

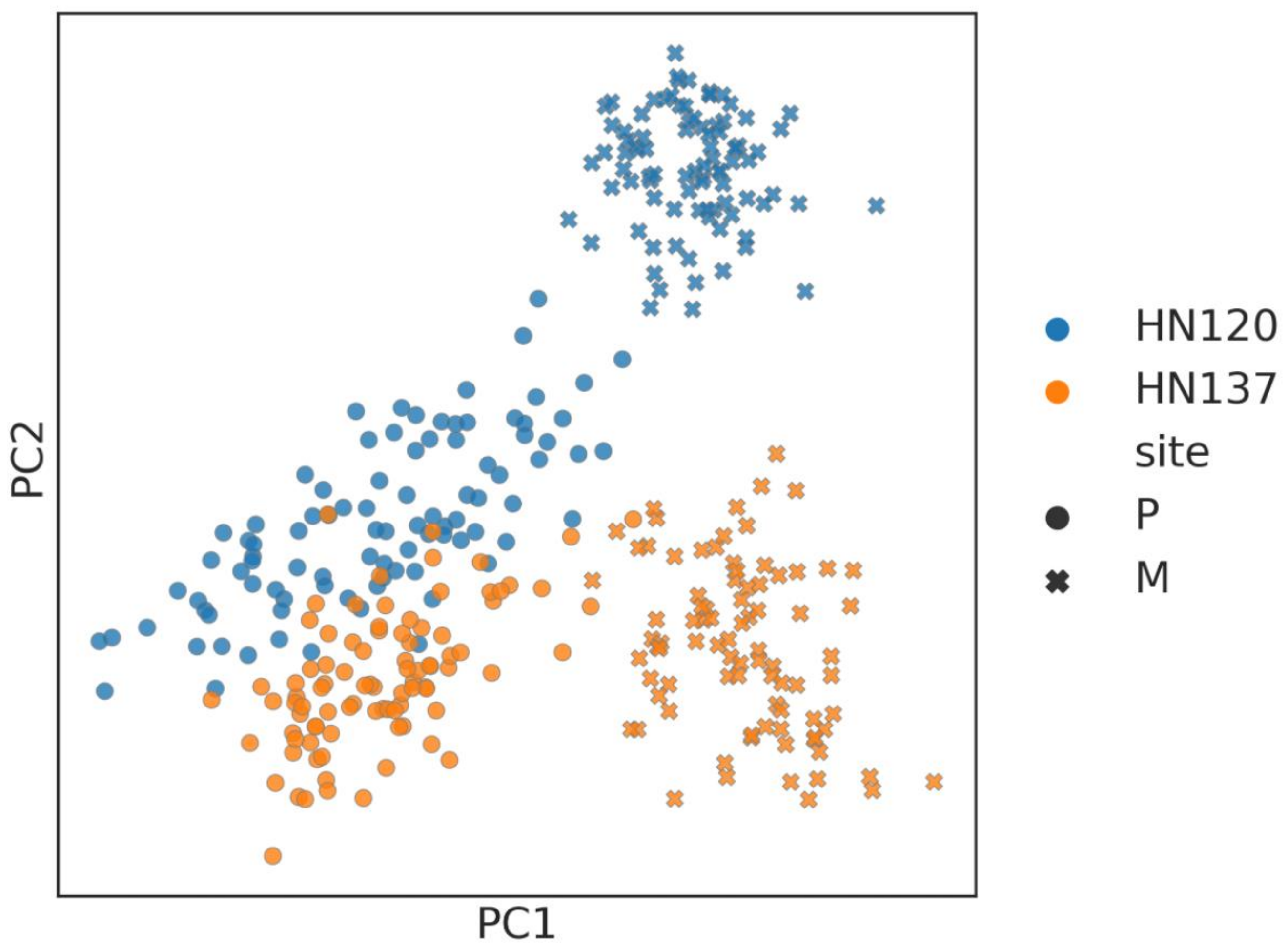

Fig S9: Transcriptomic patterns of cells from HN120 and HN137. Cells from primary tumor of HN120 and HN137 have similar transcriptomic pattern, while cells from metastatic tumor have distinct transcriptomic patterns, suggesting both inter-patient and intra-tumoral heterogeneity.

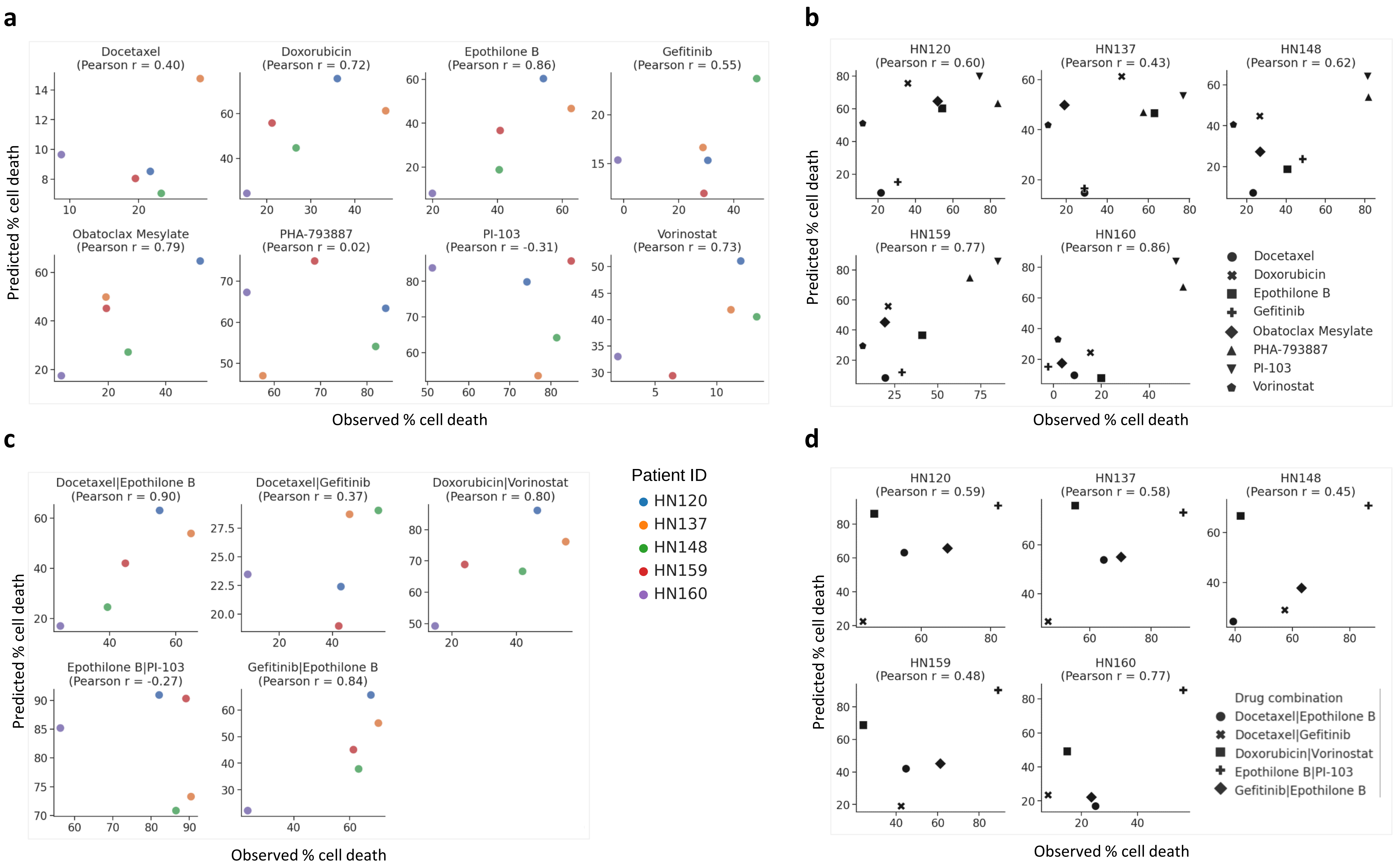

Fig S10: Detailed comparison between predicted and observed cell death percentages. Results reported are at 3-fold dilution dosage, including (**a**) monotherapy response per drug (**b**) monotherapy response per cell type (**c**) responses to drug combinations (**d**) response to drug combinations per cell type. Note that correlation values should be interpreted with caution as the number of data points is small for all plots.

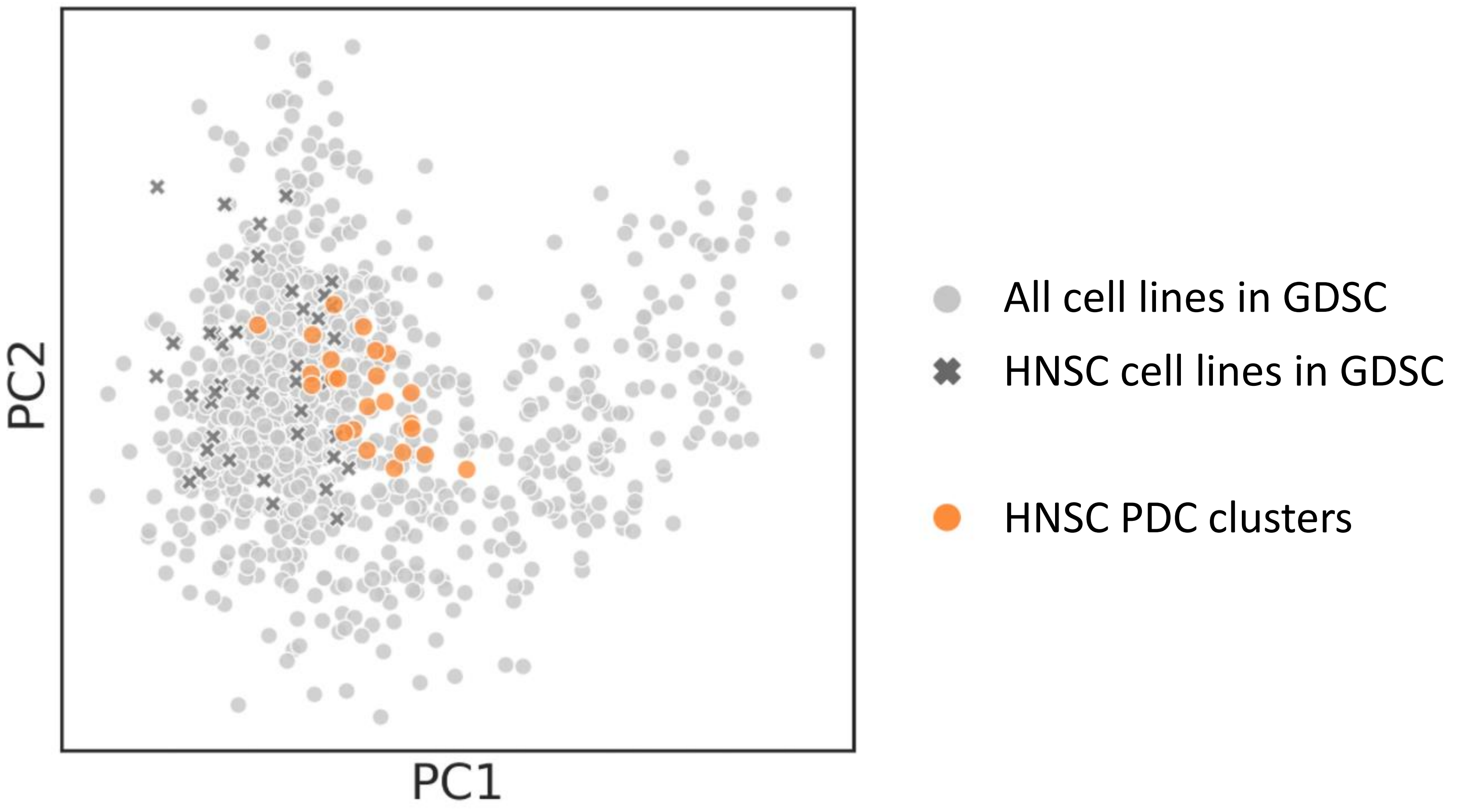

Fig S11: Pharmacogenomic space of GDSC cell lines and HNSC patient-derived cell clusters. The HNSC PDC clusters based on scRNA-seq data were found to map to a region near the GDSC head and neck cancer cell lines used for model training.

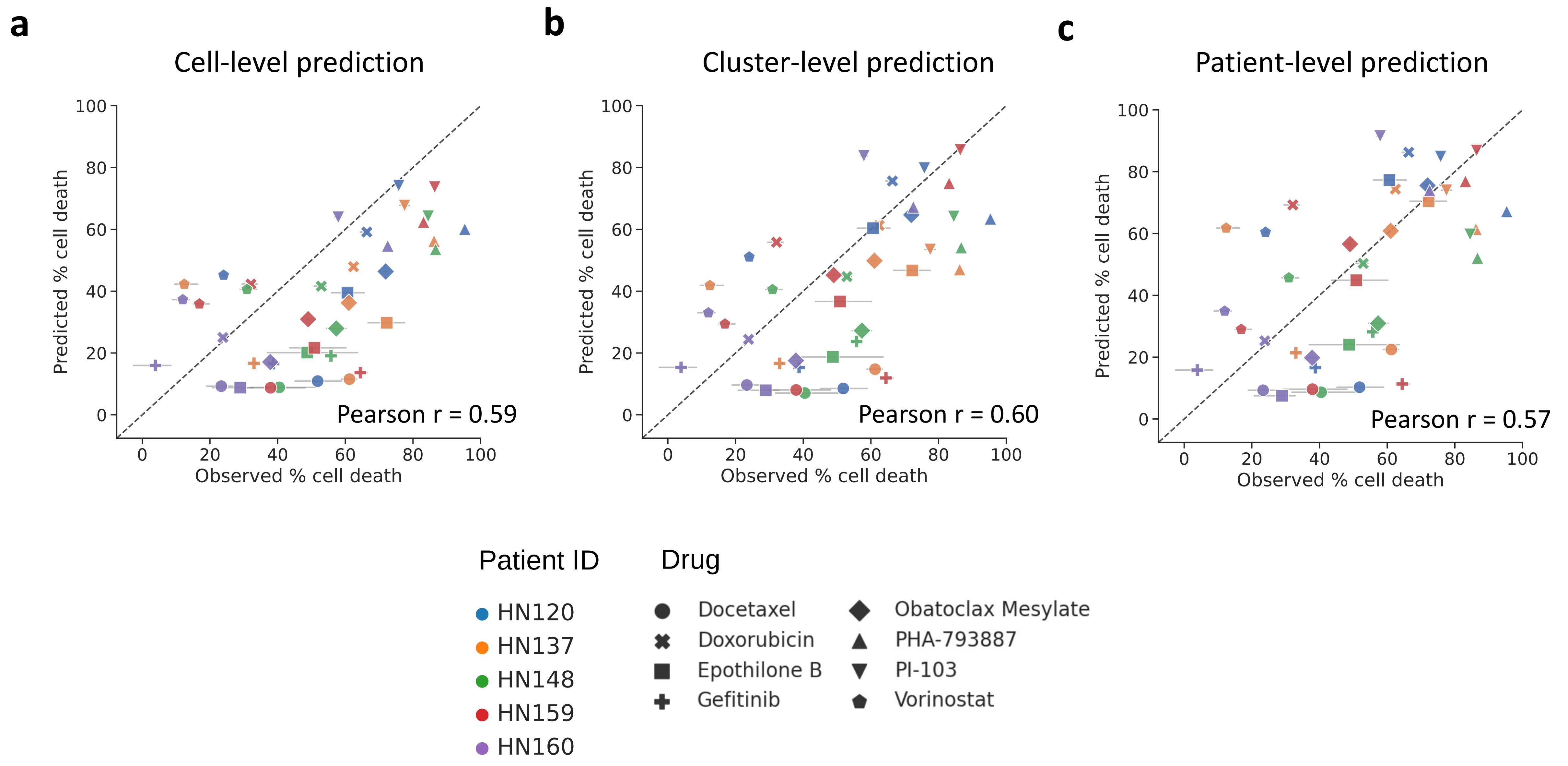

Fig S12: Comparison of observed and predicted drug response across 5 pooled PDCs and 8 drugs. Prediction based on transcriptomic profiles of (**a**) each cell combined at the patient-level, (**b**) each cell cluster combined at the patient-level, and (**c**) at the patient-level. The results reported here are based on the higher drug concentrations tested.

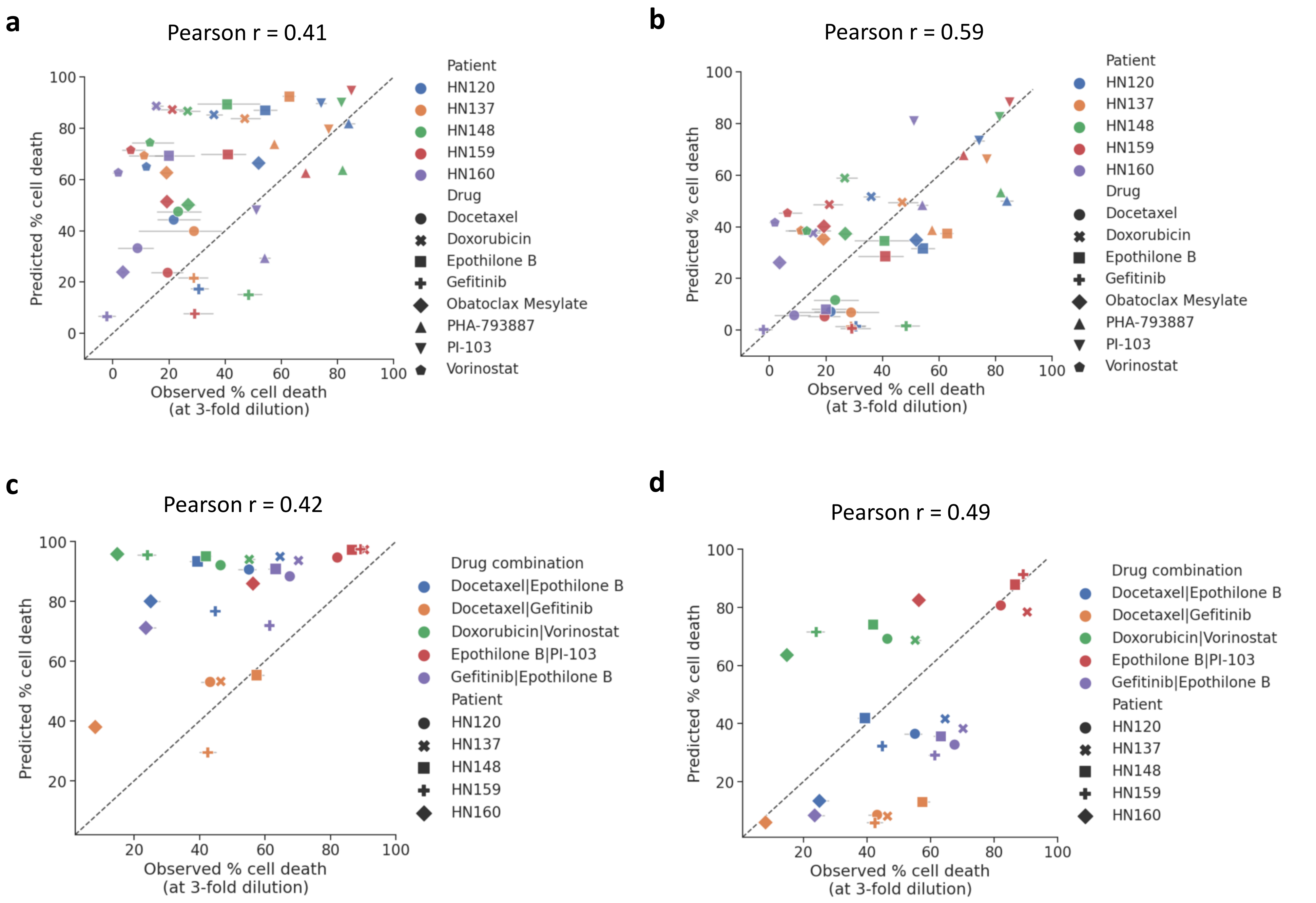

Fig S13: Predictive performance of ElasticNet and RWEN based on cell clusters. Comparison between observed and predicted cell death percentages across 8 drugs and 5 patients for RWEN (**a**) and ElasticNet (**b**). Comparison between observed and predicted cell death percentages across 5 combinatorial drugs and 5 patients for RWEN (**c**) and ElasticNet (**d**).

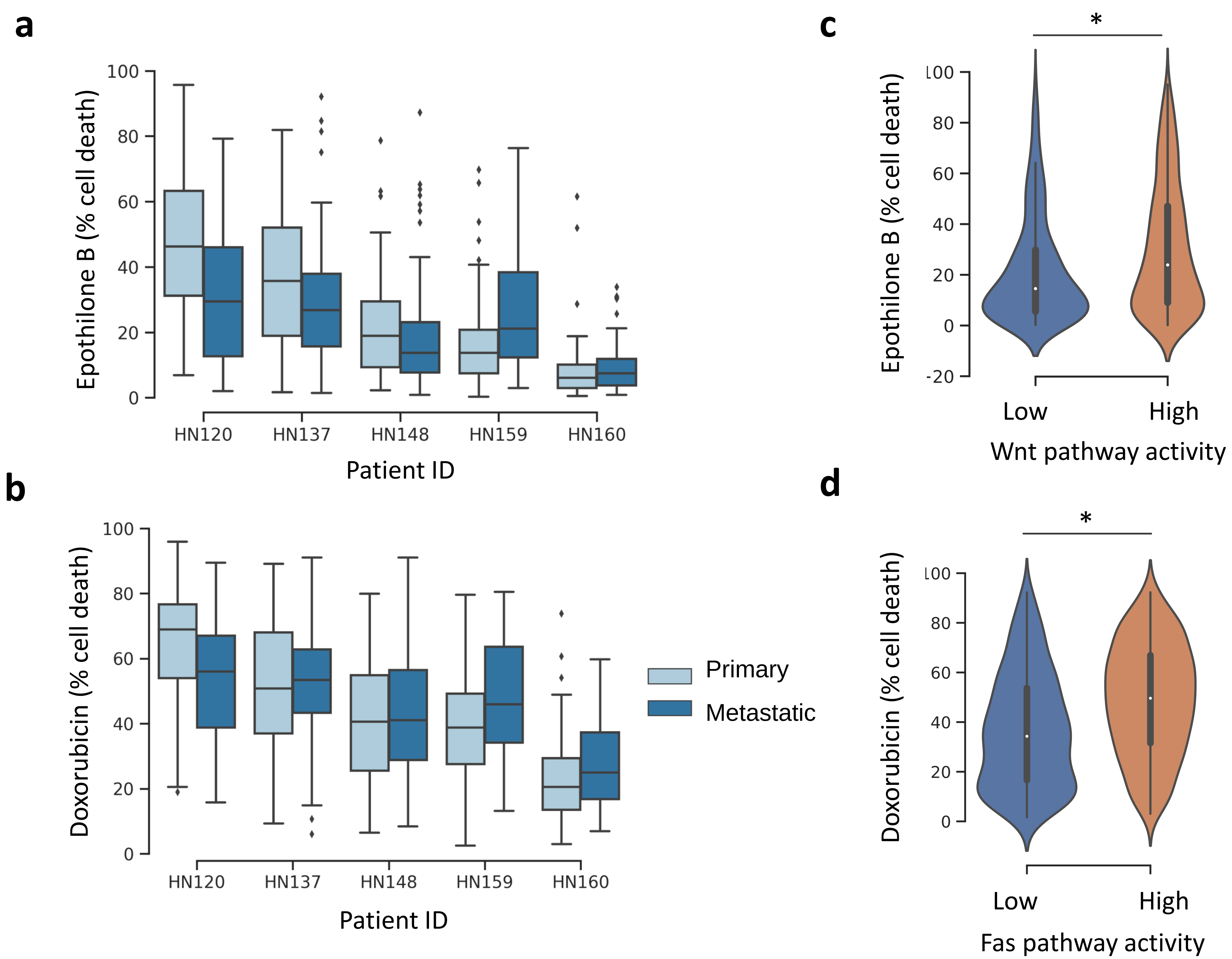

Fig S14: Comparison of drug response between tumor types and pathway activity groups. (**a-b**) Comparison of predicted drug response from matched primary and metastatic PDCs with CaDRReS-Sc. (**c-d**) Comparison of pathway activity and predicted drug response in individual cells, highlighting higher sensitivity for cells with higher activity of corresponding pathways (Wnt for Epothilone B and Fas for Doxorubicin).
